# A three-stage stochastic optimization model integrating 5G technology and UAVs for disaster management

**DOI:** 10.1007/s10898-023-01274-z

**Published:** 2023-02-24

**Authors:** Gabriella Colajanni, Patrizia Daniele, Anna Nagurney, Ladimer S. Nagurney, Daniele Sciacca

**Affiliations:** 1 Department of Mathematics and Computer Science, University of Catania, Viale Andrea Doria, 6, 95125 Catania, Italy; 2 Department of Operations and Information Management, Isenberg School of Management, University of Massachusetts, Amherst, MA 01003 USA; 3 Department of Electrical and Computer Engineering, University of Hartford, West Hartford, CT 06117 USA

**Keywords:** 5G, UAVs, Disaster management, Stochastic optimization, Network model

## Abstract

In this paper, we develop a three-stage stochastic network-based optimization model for the provision of 5G services with Unmanned Aerial Vehicles (UAVs) in the disaster management phases of: preparedness, response and recover/reconstruction. Users or devices on the ground request services of a fleet of controller UAVs in flight and the requested services are executed by a fleet of UAVs organized as a Flying Ad-Hoc Network and interconnected via 5G technology. A disaster scenario can create difficulties for the provision of services by service providers. For this reason, in the first stage, service providers make predictions about possible scenarios in the second stage. Therefore, the first stage represents the preparedness phase, the second stage represents the response phase, followed by the recovery/reconstruction phase, represented by the third stage. In each of the three stages, service providers seek to maximize the amount of services to be performed, assigning each service a priority. They also aim to, simultaneously, minimize the total management costs of requests, the transmission and execution costs of services, the costs to increase the resources of the pre-existing network and, if need be, to reduce them in the recovery/reconstruction phase. For the proposed multi-stage stochastic optimization model, we provide variational formulations for which we investigate the existence and uniqueness of the solution. Finally, a detailed numerical example is solved in order underline some of the key aspects of the model. This paper adds to the literature on the rigorous mathematical modeling of advanced technologies for disaster management.

## Introduction

5G technology, whose rollout began in 2019, can provide greater efficiency and versatility to support advanced network-based applications worldwide (see [34]). The use of 5G can bolster the digital infrastructure of companies, governments, educational institutions, non-profit organizations, and individuals (see [7]). 5G is enabling the identification and development of novel use cases that exploit sensors and interconnected devices that generate real time data for enhanced situational awareness. For example, a firm could use a 5G-based application to acquire data on the performance of its machinery to predict future maintenance needs, potentially saving money by reducing or eliminating unplanned downtime (see [6]). The COVID-19 pandemic, in turn, has demonstrated the fundamental importance of a fully-connected society with needs such as: remote learning, virtual business meetings, and working from home. To achieve these, it is essential that the connections be fast, stable and secure (see [21, 56]), all characteristics of 5G (see [2, 20]).

In parallel with the advent of 5G, the development of various technologies, such as Unmanned Aerial Vehicles (UAVs) (drones, balloons, etc.) has led to possible synergies with 5G in consumer, commercial and civil applications ( [3, 33, 47]) as well as humanitarian ones. Demand for UAV applications spans all industries as companies adopt remote monitoring and automation for fast and efficient operations. However, there are limitations that restrict how effectively UAVs can perform, with the main challenge being connectivity. Most UAVs require continuous communication with their controllers in order to ensure safety and effective operations. Traditionally, the operational range of UAVs has been limited by the range of the radio controller (RC) resulting in most of them operating at low altitudes. However, despite significant efforts to develop proprietary radio control solutions, UAV designers have been able to improve the range by only a few kilometers and only within the vision line of sight (VLOS). The advent of 4G and its subsequent progression to 5G have proven to be revolutionary. Through superior connectivity, UAVs are expected to autonomously perform complex missions, transmit and upload large amounts of high-definition data and video to the cloud, and travel much greater distances, even beyond the line of sight (BVLOS) (see [31]). The extremely low latency of 5G (see [52]) should further revolutionize the collection and transmittal of data at unprecedented speeds, the integration of artificial intelligence (AI) (see [35]), the streaming of real-time ultra-high-definition images and videos (see [49]), and enhanced air traffic intelligence.

As already noted, the fields of application of UAVs supported by 5G technology are many and diverse. Importantly, in a disaster situation, whether natural or man-made, slow-onset or sudden-onset, the collection and analysis of data in real-time are paramount and can assist in the saving of lives, the reduction of pain and suffering, and the protection and restoration of infrastructure. UAVs have emerged as powerful tools for disaster management with uses including: the video-monitoring of fires, landslides, earthquakes, and their impacts (see [55]), search and rescue, law enforcement support, oil and gas field detection, infrastructure inspections, land mapping and even deliveries of needed supplies (see [16, 40, 45, 51]).

The nature of disaster events and their diverse triggers dictate the need to introduce uncertainty of different parameters, including the occurrence of such events in rigorous mathematical models. In general, when a disaster occurs, it is plausible to assume that the physical connectivity that guarantees the availability of services may be compromised. In many disasters, it is possible to quickly restore the physical connectivity to the predisaster level. However, during many disasters a straighforward restoration of connectivity may not be possible. In fact, it may be more effective to introduce new connectivity and services in the disaster area. For example, if the disaster causes significant disruption at the ground level, the upgrading of a 4G network to a 5G one could be the most effective way to restore connectivity. An additional benefit of this type of restoration is the added availability of new services for the responders.

Since a disaster can irreversibly alter human activities, both the preparedness phase and the response phase are of fundamental importance in disaster management. Specifically, decision-makers and managers, in the disaster preparedness phase, need to consider multiple scenarios with different probabilities of occurrence so as not to be surprised in the response phase, and to be able to minimize the potential losses. The use of UAVs, organized as a fleet (FANET, Flying Ad-Hoc Network) in a 5G network could allow service providers to restore connectivity and provide much needed services in disaster areas. During a disaster, new scenarios may arise, before normalcy is restored. For these reasons, in this paper, we construct a three-stage stochastic optimization model in which each of the three stages represents a phase of disaster management: the preparedness phase, the response phase and the recovery/reconstruction phase. In terms of our model, in the first stage, representing the preparedness phase, the service provider solves a maximization problem, with the aim of maximizing the total executed 5G services (favoring the services with higher priority levels), while, simultaneously, minimizing the overall cost. In this stage, the variables, the parameters as well as the cost functions are deterministic.

In the second stage, a typical service provider evaluates several scenarios, each with different probabilities. The provider might decide to increase or decrease the capacities of the controller UAVs and whether to add new UAVs at the FANET. The aim in this stage is to maximize the quantity of executed services, while minimizing losses related to the unmet demand in services and the costs, including those due to the possible reduction in the capacities of controller UAVs. As a consequence, the actions taken by a service provider during the second stage depend on the possible scenarios and their occurrences as well as on the expected utility associated with the third stage. In the third stage, the service provider aims to maximize the quantity of executed services and has to also take into account different scenarios with different probabilities of occurrence.

Furthermore, battery life is one of the most important issues when using UAVs, since it restricts flight duration. Therefore, the service provider also seeks to reduce the power consumption of UAVs, with the aim of extending the life of their batteries (and, hence, their use) as much as possible.

The rest of the paper is organized as follows. In Sect. 2, we provide a literature review of optimization models applied to 5G networks using UAVs and of stochastic optimization, in general, and we describe our contributions in this paper. Section 3 is devoted to the description of the constrained stochastic optimization model. In Sect. 4, we provide variational formulations of the proposed optimization models, ensuring the existence of a solution. Section 5 contains a numerical example to validate the effectiveness of the proposed model and, finally, Sect. 6 is devoted to the summary and the conclusions.

## Literature review and our contributions

In this Section, we review the main bibliographic sources in the literature that in the recent past have dealt with the management and optimization of 5G networks (with and without UAVs) and relevant stochastic multi-stage optimization models.

As already mentioned, the advent of 5G technology and the related advantages, have led several researchers to tackle the problem of managing 5G networks. Addad et al. in [1] address the complexities and heterogeneities of verticals targeted by 5G systems. The authors propose and evaluate a Mixed Integer Linear Programming Problem (MILP) optimization model to tackle the complexities that arise in this problem, enabling a cost-optimal deployment of network slices allowing a mobile network operator to efficiently allocate the underlying layer resources according to its users’ requirements. In [12], a multi-tiered network-based optimization model is presented describing the provision of services via network slices of 5G-service providers, taking into account the security levels of each provider. The objective of the proposed model is to establish the optimal flows between network layers and the optimal security levels in order to maximize the providers’ profits, given by the difference between the revenues obtained by the sale of services and the rental of their resources and the costs. Numerical experiments are performed and examples solved with a new nature-inspired genetic algorithm adapted to the 5G network optimization problem. In [22], the authors provide a standardized and easy to understand Integer Linear Program (ILP) for offline mobile network slice embedding, focusing on resource allocation with a virtual node as well as link mapping. The objective of the proposed model is to maximize the weighted sum of all embedded network slices. Finally, a simple configuration is solved using SCPSolver, a Java interface for ILP which is based on the GLPK (GNU Linear Programming Kit). Xu et al. in [53] take into account the problem of the limitations of mobile devices, widely used in our daily lives. Assuming that these limitations can be reduced by enhancing the central units (CUs) in 5G into edge nodes for processing, they propose an optimization problem devoted to improving the resource utilization and load balance for all the edge nodes while protecting the privacy information and satisfying the time requirement.

The importance of using UAVs in 5G networks is emphasized in the multitude of mathematical models present in the literature. Moreover, several surveys have dealt with revising optimization models regarding the use of UAVs in 5G networks (see, for instance, [32, 50] for an extensive review on the use of drones in various applications, especially in routing problems in the context of parcel delivery and on UAVs joint optimization problems and machine learning, respectively). See [30] for a review on routing problems of two-echelon networks for a fleet of Ground Vehicles (GVs) working in collaboration with UAVs; combinations and synchronizations are modeled as Traveling Salesman Problems (TSPs), Vehicle Routing Problems (VRPs), Location Routing Problems (LRPs), Truck and Trailer Routing Problems (TTRPs) and so on (an overview of the recent literature on multimodal transportation optimization is presented in [5]). In [15], a three-tier supply chain network model is presented consisting of a fleet of UAVs organized as a FANET connecting one to another with direct wireless links, managed by a fleet of UAV controllers, whose purpose is to provide 5G network slices on demand to users and devices on the ground. The aim of this optimization model is to determine the optimal distributions of request flows. Burdakov et al. in [10], consider an optimization problem originating from optimal placement of communications relay nodes. To this purpose, the authors consider the number of unmanned vehicles (both aerial and ground vehicles), and their positions as decision variables and as the objective function they define the placement quality. In [19], the authors propose a framework to assign optimal locations to UAVs-enabled aerial relays. The proposed problem is composed of UAVs-user association and UAVs-aerial placement. The prospective problem is based on probabilistic and deterministic Line of Sight (LOS) classification, making efficient use of a city map. A low-complexity based technique is used to estimate the placement of UAVs concerning user locations. In [23], a new multi-UAV reconnaissance task allocation model is proposed. The objective function consists of the weighted sum of the total UAV consumption and the task execution time and the aim of the proposed model is to minimize it. A new heuristic algorithm, called a grouping ant colony optimization algorithm, is proposed for this new model and compared with the traditional one. The authors in [24] analyze the coordination of network-enabled UAVs that provide communication coverage to multiple mobile users on the ground. The aim of this model is to maximize the set of mobiles covered by UAVs by balancing the power consumption. Zhao et al. in [54] propose a UAV-assisted non-orthogonal multiple access (NOMA) network, in which the UAV and base station (BS) cooperate with each other to serve ground users simultaneously. The sum rate is maximized by jointly optimizing the UAV trajectory and the NOMA precoding. The proposed optimization is decomposed into two steps. First, the sum rate of the UAV-served users is maximized via alternate user scheduling and UAV trajectory with its interference to the BS-served users below a threshold. Then, the optimal NOMA precoding vectors are obtained using two schemes with different constraints. In both schemes, the non-convex optimization problems are converted into tractable ones and an iterative algorithm is designed to solve some numerical experiments. Authors in [29] present a humanitarian Coverage Path Planning (CPP) framework optimized using a homogeneous fleet of UAVs, with the aim of minimizing the sum of arrival times in each point of a certain area of interest. Using a grid-based method of cellular decomposition, authors transformed the CPP problem into a VRP and implemented some heuristics to effectively solve it (see also [25, 39, 44] for other VRPs with UAVs).

The great versatility of UAVs and the purposes for which they can be used also in 5G networks can provide decisive assistance in disaster management. The uncertainty of the occurrence of disaster events dictates the need to take into account stochastic parameters into mathematical (optimization) models. Several researchers have applied multi-level stochastic programming theory to model the different stages of disaster management (see [26] for a review of the state-of-the-art of the literature on two-stage stochastic programming in disaster management). In [8], the authors propose a two-stage stochastic programming model to plan the transportation of vital first-aid commodities to disaster-affected areas during emergency response. A multi-commodity, multi-modal network flow formulation is developed to describe the flow of material over an urban transportation network. Since it is difficult to predict the timing and magnitude of any disaster and its impact on the system, which the authors consider to be urban, resource mobilization is treated in a random manner, and the resource requirements are represented as random variables.

Daniele and Sciacca, in turn, in [17], propose a stochastic Generalized Nash Equilibrium model describing the competition among hospitals with first aid departments for patients in a disaster scenario where each hospital with a first aid department has to solve a two-stage stochastic optimization problem, one before the declaration of the disaster scenario and one after the disaster occurs, to determine the equilibrium hospitalization flows to dispatch to the other hospitals with first aid and/or to hospitals without emergency rooms in the network. Nagurney and Salarpour in [38], for the first time, propose a two-stage stochastic game theory model describing the behavior of national governments in a healthcare disaster inspired by the COVID-19 pandemic and their competition for essential medical supplies in different phases of disaster management. Noyan in [43] consider a risk-averse two-stage stochastic programming model, where is specified the conditional-value-at-risk (CVaR) as the risk measure. In particular, in this work the problem of determining the response facility locations and the inventory levels of the relief supplies at each facility in the presence of uncertainty in demand and the damage level of a disaster network is addressed. In [46], authors provide a three-stage mixed-integer stochastic programming model for disaster response planning, considering the opening of local distribution facilities, the initial allocation of supplies, and the last mile distribution of aid. The vehicles available for transportation, the state of the infrastructure and the demand for the potential beneficiaries are considered as stochastic elements. Authors in [4] present a two-stage stochastic 0–1 modeling and a related algorithmic approach for Supply Chain Management under uncertainty, whose goal consists in determining the production topology, plant sizing, product selection, product allocation among plants and vendor selection for raw materials. The objective is the maximization of the expected benefit given by the product net profit over the time horizon minus the investment depreciation and operations costs. As part of the provision of 5G services in disaster scenarios, Colajanni et al. in [14], for the first time, present a two-stage stochastic optimization model (solved with the use of variational inequality theory) for the provision of such services in a multi-tiered network, consisting of users or devices on the ground requiring services from controller UAVs in flight. Since the possible occurrence of disaster scenarios and the related uncertainty and severity could cause an unexpected and sudden increase in demand, the authors propose to optimize the management of the pre-existing and additional resources in order to maximize the total profit of service providers and, simultaneously, to minimize the expected loss related to a possible unmet demand.

The theoretical framework proposed in this work represents a natural and, yet, significant extension of the two-stage stochastic model just described. Our contributions in this paper can be summarized as follows:

- We formulate a three-stage stochastic optimization model for the provision of services in a multi-tiered network and in a disaster framework.
- The proposed model optimizes the management of the pre-existing and additional resources in order to maximize the most important (and necessary) executed 5G services, while minimizing the overall cost. The objective functions employed differ from those in the earlier noted 2-stage model. Indeed, the purpose of this article is different, as it mainly aims to maximize the 5G services provided to users making use of one of the specific characteristics of 5G: the network slicing.
- We provide variational formulations of the proposed optimization model, accompanied by a detailed numerical example.

This work adds to the literature on the mathematical modeling of advanced technology and associated services for disaster management.

## Mathematical model

In this Section, we present the mathematical model; specifically, we derive a three-stage stochastic constrained optimization model.

First, we describe the generic 5G service supply network, on which the mathematical model is based. As mentioned in Sect. 1, several types of 5G services can be requested not only by users but also by devices, both situated on the ground. For the purposes of formulating the model, it does not matter if the requests for services are made by users or by devices; therefore, we represent users or devices in the network through nodes (in the same layer), distinguishing, however, the type of requested service. The service requests are distinguished according to the type of service, and, in the context of a disaster management setting can represent, for example: video monitoring, the sharing of sensor data, assessment of infrastructure status, identification of possible victims, medical relief delivery, temperature scanning, crowd monitoring (headcounts, social distancing, lockdown enforcement), population alerting (communication through speakers), video calling, smart-traffic control, and so on. Moreover, each service requires 5G technology to work and the requests for services are managed (again via the 5G network) by some controller UAVs which act as network orchestrators and which, in turn, send the service requests to the UAVs belonging to the fleet at the highest layer, where services are actually executed. Observe that both the controller UAVs and the UAVs belonging to the fleet are owned by the service provider, which aims to provide the requested services and, at the same time, to manage its resources (i.e., its network of UAVs) in the best possible way.

Therefore, a generic network to provide 5G services consists of 3 layers (from the top to bottom): the execution layer where services are performed, the orchestration layer in which some controller UAVs manage the service requests, and the layer of requests for 5G services. Consequently, we take into account a fleet of UAVs on which 5G services are executed, the controller UAVs, which receive the service requests and send them to the UAVs belonging to the fleet, and the service requests demanded by users and devices.

As previously described, in this work we assume that, due to a certain event, the 5G service requests could increase in stage 2. To properly manage the increase in requests, we suppose that the service provider could decide if to use some additional UAVs and/or to increase the controller UAVs’ capacities; namely, the maximum number of service requests that the controller UAVs are able to manage. Thus, we consider $\hat{F}_1$ pre-existing UAVs, which are already actively part of the network, as they are in flight, included in the FANET, and $\tilde{F}_2$ additional ones which could be used, if necessary. Moreover, we denote by $\mathcal {\hat{F}}_1=\{\hat{1},\ldots ,\hat{f},\ldots ,\hat{F}_1\}$ and by $\mathcal {\tilde{F}}_2=\{\tilde{1},\ldots ,\tilde{f},\ldots ,\tilde{F}_2\}$ the sets of pre-existing and additional UAVs, respectively, and by $\mathcal {{F}}_3=\mathcal {\hat{F}}_1\cup \mathcal {\tilde{F}}_2$ the set of the union of all UAVs belonging to the fleet at the highest layer of the network. In contrast, in stage 3, after the disastrous event and the response phase are over, the 5G service requests could decrease. Hence, the provider may not use the additional UAVs belonging to the highest layer, but may also decide to reduce the capacities of the controller UAVs of the middle layer. We also consider *K* different types of services (we denote by *k* the typical one), *U* controller UAVs (we denote by *u* the typical one) and *G* users or devices on the ground (we denote by *g* the generic user or device).

**Fig. 1:**
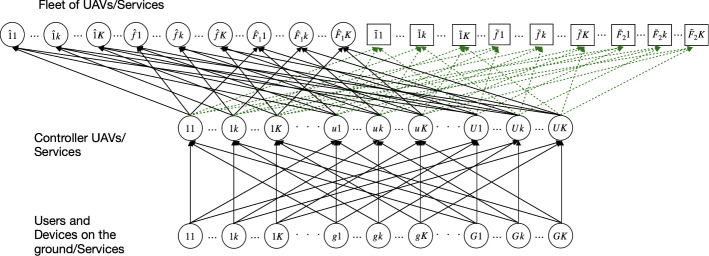
Network topology (where we denote by $1,\ldots ,k,\ldots ,K$ the services, by $\hat{1},\ldots ,\hat{f},\ldots ,\hat{F}_1$ the pre-existing UAVs, by $\tilde{1},\ldots ,\tilde{f},\ldots ,\tilde{F}_2$ the additional UAVs, by $1,\ldots ,u,\ldots ,U$ the controller UAVs and by $1,\ldots ,g,\ldots ,G$ the users and devices)

Hence, the 5G service supply network, as shown by Fig. 1, consists of:

- The combination between each UAV (which executes the 5G services) belonging to the fleet at the highest layer of the network and each service; hence, we have both the combination between each pre-existing UAV $\hat{f}=\hat{1},\ldots ,\hat{F}_1$ and each additional UAV $\tilde{f}=\tilde{1},\ldots ,\tilde{F}_2$ with each service $k=1,\ldots ,K$: $\hat{1}1,\ldots ,\hat{1}k,\ldots ,\hat{1}K$, $\ldots$, $\hat{f}1,\ldots ,\hat{f}k,\ldots ,\hat{f}K$, $\ldots$, $\hat{F}_11,\ldots ,\hat{F}_1k,\ldots ,\hat{F}_1K$ and $\tilde{1}1,\ldots ,\tilde{1}k,\ldots ,\tilde{1}K$, $\ldots$, $\tilde{f}1,\ldots ,\tilde{f}k,\ldots ,\tilde{f}K$, $\ldots$, $\tilde{F}_21,\ldots ,\tilde{F}_2k,\ldots ,\tilde{F}_2K$;
- The combination between each controller UAV in flight $u=1,\ldots ,U$ (which manages the service requests) and each service $k=1,\ldots ,K$: $11,\ldots ,1k,\ldots ,1K$, $\ldots$, $u1,\ldots ,uk,\ldots ,uK$, $\ldots$, $U1,\ldots ,Uk,\ldots ,UK$;
- The combination between each user or device on the ground $g=1,\ldots ,G$ (that requires the services) and each service $k=1,\ldots ,K$: $11,\ldots ,1k,\ldots ,1K$, $\ldots$, $g1,\ldots ,gk,\ldots ,gK$, $\ldots$, $G1,\ldots ,Gk,\ldots ,GK$.

We highlight that we used the green dashed lines for the links of the network connecting the controller UAVs with the additional UAVs (belonging to the fleet at the highest level of the network). We have chosen this format to clearly distinguish the pre-existing UAVs from the possible additional ones. Moreover, observe that not all the links are depicted, but only those connecting the corresponding services.

Note that the network presented above and the model that we propose are suitable for both 4G and 5G, but we focus on 5G since it is higher performing and because 5G provides greater bandwidth so it is possible to provide real-time video, for example, and other services in a very reduced time-frame (it is well-known that the time that it takes to transmit the data for the service is related to the bandwidth of the 5G link). Furthermore, it is assumed that, in disasters, the cell towers for 5G have not been disturbed.

**Fig. 2:**
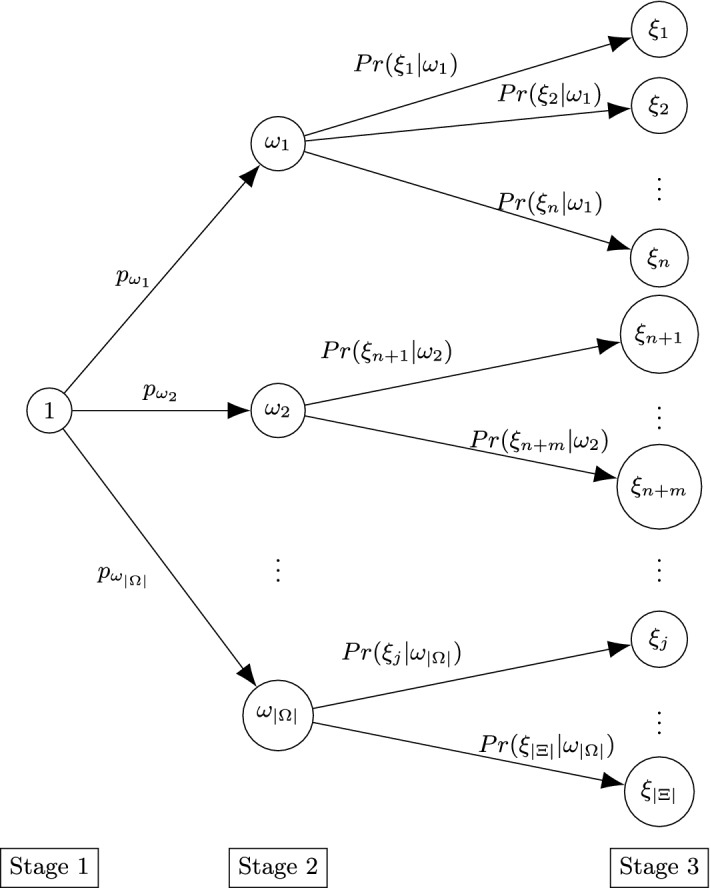
Representation of the three stages, where the circles indicate the scenarios that can occur in the Stage 1 (1), Stage 2 ($\omega _1,\omega _2,\ldots , \omega _{|\Omega |}$) and Stage 3 ($\xi _1,\xi _2,\ldots ,\xi _{|\Xi |}$), while the probabilities are shown near the links

As anticipated in the Introduction, each disastrous event can be formally described by three stages which follow one after the other. Note that, in our model, we do not consider the mitigation phase of disaster management but do handle the other three phases of preparedness, response, and recovery/reconstruction. The first stage is constituted by the normalcy conditions, in which the flows of requests for services follow a stable trend. This trend of requests may or may not undergo an abrupt change and may be more or less intense, based on the severity of the event that may occur. Therefore, the second stage is comprised of different scenarios, each of which has a probability of occurrence. Unlike the papers in the literature, among these scenarios of the second stage, we also include the scenario representing the normalcy associated with the first stage (which represents the possibility that no disastrous events will occur). Finally, after the event that takes place during the second stage, there is a return to normal and stable conditions. Obviously, if during the second stage, no changes have occurred from the previous stage, then even in stage 3 there will be no change in the demand for services; otherwise, similar to what has been described above, a scenario of stage 2 can be followed by different scenarios of stage 3 with different probabilities of occurrence and demand for services. Figure 2 shows the representation of the three stages.

Elaborating further, in the first stage, we have an initial scenario denoted by 1. After the potential disaster occurs, we find ourselves in stage 2, where different scenarios can arise. Note that one of the scenarios of stage 2 can represent the initial situation (equivalent to scenario 1 of the first stage, if no disastrous event occurs) and, as a consequence, in this case scenario 1 still occurs even at stage 3. Furthermore, it could happen that some scenarios in stage 3 are the same as some in stage 2. Moreover, it is possible that scenarios (in stage 3) coming from different scenarios of stage 2, are equivalent. For the sake of simplicity, we have decided to keep nodes representing the same scenarios separate (as distinct scenarios with the same characteristics).

The proposed mathematical model allows the service provider to maximize the quantity of provided services (favoring the services with higher priority; that is, the services considered most important and necessary) and to best manage its UAVs (minimizing the overall cost), determining for each 5G service *k* the following:

- The optimal distribution flows of data that each controller UAV *u* has to receive (and to manage) from each user or device *g*;
- The optimal distribution flows of the 5G service data that each controller UAV *u* has to send to each pre-existing UAV $\hat{f}$; namely, the quantities of services that each pre-existing UAV has to execute;
- The optimal distribution flows of the 5G service data that each controller UAV *u* has to send to each additional UAV $\tilde{f}$ and whether it is appropriate to use the additional UAVs; namely, the quantities of services that each additional UAV has to execute;
- If and how much additional capacity to add to each controller UAV *u*;
- If and how much capacity to reduce to each controller UAV *u*.

Therefore, denoting by $\Omega$ and $\Xi$ the sets of all the scenarios that could occur in the second and third stage, respectively, and denoting by $\omega \in \Omega$ and $\xi \in \Xi$ the typical scenarios, we report the variables of the model in Table 1 (note that we have reported the stage and scenario through the superscripts of the variables).

**Table 1:** Variables definition

Notation	Description
$x_{guk}^1$, $x_{guk}^{2\omega }$, $x_{guk}^{3\xi }$	Amount of data to transmit by each user or device *g* to each controller UAV *u*, associated with the service *k* in stage 1, in stage 2 when scenario $\omega \in \Omega$ occurs, in stage 3 when scenario $\xi \in \Xi$ occurs, respectively
$y_{u\hat{f}k}^1$, $y_{u\hat{f}k}^{2\omega }$, $y_{u\hat{f}k}^{3\xi }$	Amount of data to transmit by each controller UAV *u* to each pre-existing UAV $\hat{f}$, associated with the service *k* in stage 1, in stage 2 when scenario $\omega \in \Omega$ occurs, in stage 3 when scenario $\xi \in \Xi$ occurs, respectively
$y_{u\tilde{f}k}^1$, $y_{u\tilde{f}k}^{2\omega }$, $y_{u\tilde{f}k}^{3\xi }$	Amount of data to transmit by each controller UAV *u* to each additional UAV $\tilde{f}$, associated with the service *k* in stage 1, in stage 2 when scenario $\omega \in \Omega$ occurs, in stage 3 when scenario $\xi \in \Xi$ occurs, respectively
$\gamma _{u}^1$, $\gamma _{u}^{2\omega }$	The additional capacity of controller UAV *u*, in stage 1 and in stage 2 when scenario $\omega \in \Omega$ occurs, respectively
$\delta _{u}^{2\omega }$, $\delta _{u}^{3\xi }$	The reduced capacity of controller UAV *u*, in stage 2 when scenario $\omega \in \Omega$ occurs and in stage 3 when scenario $\xi \in \Xi$ occurs, respectively

Moreover, we denote the vectors of variables as follows:

$$\begin{aligned} X^1= & {} (x_{guk}^1)_{\begin{array}{c} g=1,\ldots ,G \\ u=1,\ldots ,U \\ k=1,\ldots ,K \end{array}},\;X^2=(x_{guk}^{2\omega })_{\begin{array}{c} g=1,\ldots ,G \\ u=1,\ldots ,U \\ k=1,\ldots ,K \\ \omega \in \Omega \end{array}},\;X^3=(x_{guk}^{3\xi })_{\begin{array}{c} g=1,\ldots ,G \\ u=1,\ldots ,U \\ k=1,\ldots ,K \\ \xi \in \Xi \end{array}};\\ \hat{Y}^1= & {} (y_{u\hat{f}k}^1)_{\begin{array}{c} u=1,\ldots ,U \\ \hat{f}\in \mathcal {\hat{F}}_1 \\ k=1,\ldots ,K \end{array}},\;\hat{Y}^2=(y_{u\hat{f}k}^{2\omega })_{\begin{array}{c} u=1,\ldots ,U \\ \hat{f}\in \mathcal {\hat{F}}_1 \\ k=1,\ldots ,K \\ \omega \in \Omega \end{array}},\;\hat{Y}^3=(y_{u\hat{f}k}^{3\xi })_{\begin{array}{c} u=1,\ldots ,U \\ \hat{f}\in \mathcal {\hat{F}}_1 \\ k=1,\ldots ,K \\ \xi \in \Xi \end{array}};\\ \tilde{Y}^1= & {} (y_{u\tilde{f}k}^1)_{\begin{array}{c} u=1,\ldots ,U \\ \tilde{f}\in \mathcal {\tilde{F}}_2 \\ k=1,\ldots ,K \end{array}},\;\tilde{Y}^2=(y_{u\tilde{f}k}^{2\omega })_{\begin{array}{c} u=1,\ldots ,U \\ \tilde{f}\in \mathcal {\tilde{F}}_2 \\ k=1,\ldots ,K \\ \omega \in \Omega \end{array}},\;\tilde{Y}^3=(y_{u\tilde{f}k}^{3\xi })_{\begin{array}{c} u=1,\ldots ,U \\ \tilde{f}\in \mathcal {\tilde{F}}_2 \\ k=1,\ldots ,K \\ \xi \in \Xi \end{array}};\\ \Gamma ^1= & {} (\gamma _u^1)_{u=1,\ldots ,U},\;\Gamma ^2=(\gamma _u^{2\omega })_{\begin{array}{c} u=1,\ldots ,U \\ \omega \in \Omega \end{array}};\quad \Delta ^2=(\delta _u^{2\omega })_{\begin{array}{c} u=1,\ldots ,U \\ \omega \in \Omega \end{array}},\;\Delta ^3=(\delta _u^{3\xi })_{\begin{array}{c} u=1,\ldots ,U \\ \xi \in \Xi \end{array}}. \end{aligned}$$

Let:

- $R_{gk}^{1}$, $R_{gk}^{2\omega }$ and $R_{gk}^{3\xi }$ be the demands for service *k* requested by user or device on the ground *g*, in stage 1, in stage 2 when scenario $\omega \in \Omega$ occurs, in stage 3 when scenario $\xi \in \Xi$ occurs, respectively;
- $D_k$ be the amount of data that must be transmitted associated with a unit of service *k*;
- $\rho _{k}^{1}$, $\rho _{k}^{2\omega }$ and $\rho _{k}^{3\xi }$ be the priority levels for the execution of service *k*, in stage 1, in stage 2 when scenario $\omega \in \Omega$ occurs, in stage 3 when scenario $\xi \in \Xi$ occurs, respectively (such priority levels indicate which services are most needed from a disaster victim perspective); moreover, in the disaster application domain, the controller UAVs assume an important role related to the prioritization (otherwise, a device, such as a cell phone, might directly link with a UAV);
- $\overline{S}_u$ be the maximum capacity of controller UAV *u*, that is the maximum number of requests that the controller UAV *u* is able to manage (observe that the provider could increase this maximum capacity);
- $s_k$ be the computational space used to execute a unit of service *k*; since a UAV can execute several services at the same time, $s_k$ can also be understood as a portion of resource used by a unit of service *k*;
- $S_f$ be the maximum computational space (used to execute all the requested services) on the UAV *f* belonging to the fleet at the highest layer of the network;
- $S_{fk}$ be a *specificity parameter* that is equal to 0 if the UAV *f* belonging to the fleet cannot execute service *k* (perhaps because it is not equipped with a certain type of sensor or video technology, etc.) and 1 otherwise;
- $\overline{B}^{1}$, $\overline{B}^{2\omega }$ and $\overline{B}^{3\xi }$ be the budgets that the service provider can use in stage 1, in stage 2 when scenario $\omega \in \Omega$ occurs and in stage 3 when scenario $\xi \in \Xi$ occurs, respectively;
- $\overline{\gamma }^{1}_u$ and $\overline{\gamma }^{2\omega }_u$ be the maximum additional capacities that the provider can decide to add at the controller UAV *u*, in stage 1 and in stage 2 when scenario $\omega \in \Omega$ occurs, respectively.

As mentioned above, the objective is to maximize the quantity of executed 5G services, favoring the services with higher priority levels, and to best manage the provider’s UAVs, minimizing the overall cost. The latter is given by the transmission, execution and management costs, the costs to add new UAVs to the superior fleet and the costs to increase or reduce the capacities of the controller UAVs. Hence, we denote by:

- $c_{gu}^{1}$, $c_{gu}^{2\omega }$ and $c_{gu}^{3\xi }$ the costs due to the transmission of service data from user or device *g* to the controller UAV *u*, in stage 1, in stage 2 when scenario $\omega \in \Omega$ occurs and in stage 3 when scenario $\xi \in \Xi$ occurs, respectively; and we assume that such costs are a function of the amount of data transmitted from *g* to *u*, for all services: $c_{gu}^{1}\left( \sum _{k=1}^K x_{guk}^{1}\right)$, $c_{gu}^{2\omega }\left( \sum _{k=1}^K x_{guk}^{2\omega }\right)$ and $c_{gu}^{3\xi }\left( \sum _{k=1}^K x_{guk}^{3\xi }\right) ,\;\forall g=1,\ldots ,G,\;\forall u=1,\ldots ,U,\;\forall \omega \in \Omega ,\; \forall \xi \in \Xi$;
- $c_{uf}^{1}$, $c_{uf}^{2\omega }$ and $c_{uf}^{3\xi }$ the costs due to the transmission of service data from the controller UAV *u* to the pre-existing or additional UAV *f* belonging to the fleet, in stage 1, in stage 2 when scenario $\omega \in \Omega$ occurs and in stage 3 when scenario $\xi \in \Xi$ occurs, respectively; and we assume that such costs are function of the amount of data transmitted from *u* to *f*, for all services: $c_{uf}^{1}\left( \sum _{k=1}^K y_{ufk}^{1}\right)$, $c_{uf}^{2\omega }\left( \sum _{k=1}^K y_{ufk}^{2\omega }\right)$ and $c_{uf}^{3\xi }\left( \sum _{k=1}^K y_{ufk}^{3\xi }\right) ,\;\forall u=1,\ldots ,U,\;\forall f\in \mathcal {F}_3,\;\forall \omega \in \Omega ,\; \forall \xi \in \Xi$;
- $c_{f}^{(E),1}$, $c_{f}^{(E),2\omega }$ and $c_{f}^{(E),3\xi }$ the execution costs of services to the pre-existing or additional UAV *f* belonging to the fleet, in stage 1, in stage 2 when scenario $\omega \in \Omega$ occurs and in stage 3 when scenario $\xi \in \Xi$ occurs, respectively; and we assume that such costs are a function of the total amount of executed services: $c_{f}^{(E),1}\left( \sum _{u=1}^U\sum _{k=1}^K y_{ufk}^{1}\right)$, $c_{f}^{(E),2\omega }\left( \sum _{u=1}^U\sum _{k=1}^K y_{ufk}^{2\omega }\right)$ and $c_{f}^{(E),3\xi }\left( \sum _{u=1}^U\sum _{k=1}^K y_{ufk}^{3\xi }\right) ,\;\forall f\in {\mathcal {F}}_3,\;\forall \omega \in \Omega ,\; \forall \xi \in \Xi$;
- $c_{u}^{(E),1}$, $c_{u}^{(E),2\omega }$ and $c_{u}^{(E),3\xi }$ the management costs of service requests to the controller UAV *u*, in stage 1, in stage 2 when scenario $\omega \in \Omega$ occurs and in stage 3 when scenario $\xi \in \Xi$ occurs, respectively; and we assume that such costs are a function of the received service requests and the capacities of *u* (that depend on the increased or reduced capacities):
  $$\begin{aligned}{} & {} c_{u}^{(E),1}\left( X_{u}^{1}, \gamma _u^1\right) , \,\,c_{u}^{(E),2\omega }\left( X_{u}^{2\omega },\gamma _u^1,\gamma _u^{2\omega },\delta _u^{2\omega }\right) \hbox { and }\\{} & {} \quad c_{u}^{(E),3\xi }\left( X_{u}^{3\xi },\gamma _u^1,\gamma _u^{2\omega },\delta _u^{2\omega },\delta _u^{3\xi }\right) , \,\,\forall u=1,\ldots ,U,\;\forall \omega \in \Omega ,\; \forall \xi \in \Xi , \end{aligned}$$
  where we denote by $$X_{u}^{1}=\left( x_{guk}^{1}\right) _{\begin{array}{c} g=1,\ldots ,G\\ k=1,\ldots ,K \end{array}}$$, $$X_{u}^{2\omega }=\left( x_{guk}^{2\omega }\right) _{\begin{array}{c} g=1,\ldots ,G\\ k=1,\ldots ,K \end{array}}$$ and $$X_{u}^{3\xi }=\left( x_{guk}^{3\xi }\right) _{\begin{array}{c} g=1,\ldots ,G\\ k=1,\ldots ,K \end{array}},$$ $\forall u=1,\ldots ,U,\;\forall \omega \in \Omega ,\; \forall \xi \in \Xi$. Particularly, we assume that such costs depend on the total amount of received service requests, that is on the sums $\sum _{g=1}^G\sum _{k=1}^K x_{guk}^{1}$, $\sum _{g=1}^G\sum _{k=1}^K x_{guk}^{2\omega }$ and $\sum _{g=1}^G\sum _{k=1}^K x_{guk}^{3\xi }$, respectively. Therefore, we will use the following notation later on:
  $$\begin{aligned}{} & {} c_{u}^{(E),1}\left( \sum _{g=1}^G\sum _{k=1}^K x_{guk}^{1}, \gamma _u^1\right) , \,\,c_{u}^{(E),2\omega }\left( \sum _{g=1}^G\sum _{k=1}^K x_{guk}^{2\omega },\gamma _u^1,\gamma _u^{2\omega },\delta _u^{2\omega }\right) \hbox { and }\\{} & {} \quad c_{u}^{(E),3\xi }\left( \sum _{g=1}^G\sum _{k=1}^K x_{guk}^{3\xi },\gamma _u^1,\gamma _u^{2\omega },\delta _u^{2\omega },\delta _u^{3\xi }\right) , \,\,\forall u=1,\ldots ,U,\;\forall \omega \in \Omega ,\; \forall \xi \in \Xi ; \end{aligned}$$
- $c_{\tilde{f}}^{1}$, $c_{\tilde{f}}^{2\omega }$ and $c_{\tilde{f}}^{3\xi }$ the costs due to add new UAVs to the fleet at the highest layer of the network, in stage 1, in stage 2 when scenario $\omega \in \Omega$ occurs and in stage 3 when scenario $\xi \in \Xi$ occurs, respectively; and we assume that such costs are a function of the total amount of executed services: $c_{\tilde{f}}^{1}\left( \sum _{u=1}^U\sum _{k=1}^K y_{u\tilde{f}k}^{1}\right)$, $c_{\tilde{f}}^{2\omega }\left( \sum _{u=1}^U\sum _{k=1}^K y_{u\tilde{f}k}^{2\omega }\right)$ and $c_{\tilde{f}}^{3\xi }\left( \sum _{u=1}^U\sum _{k=1}^K y_{u\tilde{f}k}^{3\xi }\right) ,$ $\forall \tilde{f}\in \mathcal {\tilde{F}}_2,\;\forall \omega \in \Omega ,\; \forall \xi \in \Xi$;
- $c_{u}^{1}$ and $c_{u}^{2\omega }$ the costs due to increase the capacities of controller UAV *u*, in stage 1 and in stage 2 when scenario $\omega \in \Omega$ occurs, respectively; and we assume that such costs are a function of the additional capacities: $c_{u}^{1}\left( \gamma _{u}^{1}\right)$ and $c_{u}^{2\omega }\left( \gamma _{u}^{2\omega }\right) ,\;\forall u=1,\ldots ,U,\;\forall \omega \in \Omega$;
- $c_{u}^{(D),2\omega }$ and $c_{u}^{(D),3\xi }$ the costs due to reduce the capacities of controller UAV *u*, in stage 2 when scenario $\omega \in \Omega$ occurs and in stage 3 when scenario $\xi \in \Xi$ occurs, respectively; and we assume that such costs are a function of the reduced capacities: $c_{u}^{(D),2\omega }\left( \delta _{u}^{2\omega }\right)$ and $c_{u}^{(D),3\xi }\left( \delta _{u}^{3\xi }\right) ,\;\forall u=1,\ldots ,U,\;\forall \omega \in \Omega ,\; \forall \xi \in \Xi$.

We highlight that in the first stage the provider can establish if, in anticipation of the event that may occur in the second stage, it is convenient to use additional UAVs and additional capacities, while the latter cannot be reduced. Therefore, introducing the following vectors:

$$\begin{aligned} \chi ^2=(X^2,\hat{Y}^2,\tilde{Y}^2, \Gamma ^1,\Gamma ^2,\Delta ^2), \quad \chi ^3=(X^3,\hat{Y}^3,\tilde{Y}^3, \Gamma ^1,\Gamma ^2,\Delta ^2, \Delta ^3), \end{aligned}$$

the service provider is faced with the following three-stage stochastic optimization model, in which it seeks to maximize the total executed 5G services and to minimize the overall cost:

1 $$\begin{aligned} \begin{aligned}&\text{ Max }\left\{ {\alpha _1}\sum _{u=1}^U\sum _{f \in {\mathcal {F}}_{3}}\sum _{k=1}^K{\rho ^1_{k}y_{ufk}^1}-{\alpha _2 } \left[ \sum _{g=1}^G\sum _{u=1}^U c^1_{gu}\left( \sum _{k=1}^K{x^1_{guk}} \right) + \sum _{u=1}^U\sum _{f \in {\mathcal {F}}_{3}} c^1_{uf}\left( \sum _{k=1}^K{y^1_{ufk}} \right) \right. \right. \\&\quad \left. + \sum _{{f} \in {{\mathcal {F}}}_3} {c_{{f}}^{(E),1}\left( \sum _{u=1}^U\sum _{k=1}^K{y^1_{u{f}k}} \right) }+ \sum _{u=1}^U {c_{u}^{(E),1}\left( \sum _{g=1}^G\sum _{k=1}^K{x^1_{guk},\gamma _u^1 } \right) }\right. \\&\quad \left. \left. + \sum _{\tilde{f}\in \tilde{{\mathcal {F}}}_2}{c^1_{\tilde{f}}\left( \sum _{u=1}^U\sum _{k=1}^K y^1_{u\tilde{f}k}\right) } +\sum _{u=1}^U c_u^1(\gamma _u^1)\right] +{\mathbb {E}}_{\Omega }[P^2(\chi ^2,\omega )+{\mathbb {E}}_{\Xi }[P^3(\chi ^3,\omega ,\xi )]]\right\} \end{aligned} \end{aligned}$$

subject to:

2 $$\begin{aligned}{} & {} \sum _{u=1}^U x^1_{guk}{\ge } R^1_{gk}\cdot D_k \quad \forall g=1,\ldots ,G,\; \forall k=1,\ldots ,K, \end{aligned}$$

3 $$\begin{aligned}{} & {} \sum _{k=1}^K\sum _{g=1}^G x^1_{guk} \le \overline{S}_{u}{+ \gamma _u^1} \quad \forall u=1,\ldots ,U, \end{aligned}$$

4 $$\begin{aligned}{} & {} \sum _{\hat{f}\in \hat{{\mathcal {F}}}_1}y^1_{u\hat{f}k}+\sum _{\tilde{f}\in \tilde{{\mathcal {F}}}_2}y^1_{u\tilde{f}k} \le \sum _{g=1}^G x^1_{guk} \quad \forall u=1,\ldots ,U,\; \forall k=1,\ldots ,K, \end{aligned}$$

5 $$\begin{aligned}{} & {} \sum _{u=1}^U\sum _{k=1}^K s_k y^1_{ufk} \le S_{f} \quad \forall f\in {\mathcal {F}}_{3}, \end{aligned}$$

6 $$\begin{aligned}{} & {} \sum _{u=1}^U y^1_{ufk} \le M\cdot S_{fk} \quad \forall f\in {\mathcal {F}}_{3},\; \forall k=1,\ldots ,K, \end{aligned}$$

7 $$\begin{aligned}{} & {} \sum _{\tilde{f}\in \tilde{{\mathcal {F}}}_{2}} {c^1_{\tilde{f}}\left( \sum _{u=1}^U\sum _{k=1}^K y^1_{u\tilde{f}k}\right) } +\sum _{u=1}^Uc_u^1(\gamma _{u}^1)\le \overline{B}^1, \end{aligned}$$

8 $$\begin{aligned}{} & {} \gamma _u^1\le \overline{\gamma }_u^1, \quad \forall u=1,\ldots ,U, \end{aligned}$$

9 $$\begin{aligned}{} & {} x^1_{guk},y^1_{u\hat{f}k}, y^1_{u\tilde{f}k}, \gamma _u^1 \in {\mathbb {R}}_+, \forall g,\;\forall u,\;\forall \hat{f}\in \hat{{\mathcal {F}}}_1,\; \forall \tilde{f}\in \tilde{{\mathcal {F}}}_2,\; \forall k. \end{aligned}$$

The constrained optimization problem (1)–(9) is a multi-objective programming problem; indeed, the model involves more than one objective function which must be maximized. We used the weighted sum method that combines and converts all the objective functions into a single-objective composite function using the weighted sum with $\alpha _1$ and $\alpha _2$.

The objective function (1) consists of three main terms. The first term, multiplied by the weight $\alpha _1$, represents the amount of 5G services provided that, especially in a disaster situation, must be maximized. Specifically, observe that the quantity of each executed service (namely, the quantity of data that all the controller UAVs sent to each pre-existing and additional UAVs for the execution, $\sum _{u=1}^U\sum _{f \in {\mathcal {F}}_{3}}{y_{ufk}^1}$) is multiplied by $\rho ^1_{k}$, the priority level for the execution of service *k*. The second term of the objective function, multiplied by the weight $\alpha _2$, represents the overall cost. The minus sign assures the minimization of this term. More specifically, such a total cost is given by the sum of all the following costs:

- $\sum _{g=1}^G\sum _{u=1}^U c^1_{gu}\left( \sum _{k=1}^K{x^1_{guk}} \right)$, the transmission costs of service data between the users and devices on the ground and the controller UAVs;
- $\sum _{u=1}^U\sum _{f \in {\mathcal {F}}_{3}} c^1_{uf}\left( \sum _{k=1}^K{y^1_{ufk}} \right)$, the transmission costs of service data between the controller UAVs and the UAVs belonging to the fleet;
- $\sum _{{f} \in {{\mathcal {F}}}_3} {c_{{f}}^{(E),1}\left( \sum _{u=1}^U\sum _{k=1}^K{y^1_{u{f}k}} \right) }$, the execution costs for each UAV belonging to the fleet at the highest level of the network;
- $\sum _{u=1}^U {c_{u}^{(E),1}\left( \sum _{g=1}^G\sum _{k=1}^K{x^1_{guk},\gamma _u^1} \right) }$, the management costs for each controller UAV;
- $\sum _{\tilde{f}\in \tilde{{\mathcal {F}}}_2}{c^1_{\tilde{f}}\left( \sum _{u=1}^U\sum _{k=1}^K y^1_{u\tilde{f}k}\right) }$, the costs to add the new UAVs;
- $\sum _{u=1}^U c_u^1(\gamma _u^1)$, the costs to add further capacity to the controller UAVs.

Note that, in this paper, we also account for one of the most important issues in using UAVs (such as drones), the lifetime of batteries, which restricts the flight duration since the proposed model aims at minimizing the execution and management costs of each UAV.

The last term of the objective function (1) represents the expected value of the objective function of service provider in the second and third stage. Assuming a discrete probability distribution, and denoting by $p_{\omega }$ the probability that the scenario $\omega$ occurs in stage 2 and by $Pr(\xi |\omega )$ the conditional probability of $\xi$ assuming that the scenario $\omega$ has already occurred, the expected objective function of service provider in the second stage ${\mathbb {E}}_{\Omega }[P^2(\chi ^2,\omega )+{\mathbb {E}}_{\Xi }[P^3(\chi ^3,\omega ,\xi )]]$ can be written as follows:

$$\begin{aligned} \begin{aligned}&{\mathbb {E}}_{\Omega }[P^2(X^2,\hat{Y}^2,\tilde{Y}^2, \Gamma ^1,\Gamma ^2,\Delta ^2,\omega )+{\mathbb {E}}_{\Xi }[P^3(\chi ^3,\omega ,\xi )]]\\&\quad =\sum _{\omega \in \Omega }p_{\omega }\left( P^2(X^2,\hat{Y}^2,\tilde{Y}^2, \Gamma ^1,\Gamma ^2,\Delta ^2,\omega )+{\mathbb {E}}_{\Xi }[P^3(\chi ^3,\omega ,\xi )]\right) \\&\quad = \sum _{\omega \in \Omega }\Bigg [p_{\omega }\bigg ( P^2(X^2,\hat{Y}^2,\tilde{Y}^2, \Gamma ^1,\Gamma ^2,\Delta ^2,\omega )\\&\quad \quad \quad +\sum _{\xi \in \Xi }Pr(\xi |\omega )\left( P^3(X^3,\hat{Y}^3,\tilde{Y}^3, \Gamma ^1,\Gamma ^2,\Delta ^2, \Delta ^3,\omega ,\xi )\right) \bigg )\Bigg ]. \end{aligned} \end{aligned}$$

We will explain $P^2(\chi ^2,\omega )$ and $P^3(\chi ^3,\omega ,\xi )$ in detail later.

Constraint (2) states that, in stage 1, the amount of transmitted data for service *k* that all the controller UAVs receive by user or device *g* must be at least equal to the total amount of requested data, given by the demand for *k* by *g* multiplied by the amount of data that must be transmitted associated with a unit of requested service *k*. Constraint (3) guarantees that the maximum capacity of each controller UAV *u* is not exceeded. Note that such a maximum capacity could be increased, if convenient. The conservation law, corresponding to each controller UAV *u* and each service *k*, is represented by constraint (4), according to which the quantity of 5G service data transmitted by *u* to the pre-existing and additional UAVs at the highest layer of the network (for the execution) must be less than or equal to the quantity of 5G service data that *u* has received from all the users and devices. The maximum computational space (used to execute all the requested services) of each UAV *f* belonging to the fleet is not exceeded, as established by constraint (5). We assume that a specific service may require a specific type of UAV (such as one with a certain type of sensor or video technology, etc.). Therefore, not every UAV can perform any requested service function as established by constraint (6), where *M* is a very big number. Indeed, if the specificity parameter $S_{fk}=0$; namely the UAV *f* cannot execute service *k*, then constraint (6) assures that $\sum _{u=1}^Uy_{ufk}^1 \le 0$; namely, there is no flow of data, associated with the service *k*, transmitted to the UAV *f*. On the contrary, if the specificity parameter $S_{fk}=1$, we have no restrictions on the flow of data transmitted to the UAV *f*. Constraint (7) means that, in the first stage, the sum of costs needed to use additional UAVs to execute the 5G services and to increase the capacities of controller UAVs cannot exceed $\overline{B}^1$, the maximum budget available to the provider. Moreover, constraint (8) must be verified; that is, the capacity constraint, according to which it is possible to add only a limited quantity of capacity on the controller UAV *u*. Finally, constraints (9) are constraints of nonnegativity of the variables.

The optimal solution in the second stage, in turn, is determined as the solution to the following second stage stochastic maximization problem:

10 $$\begin{aligned} \begin{aligned}&\text{ Max }\left\{ {\alpha _1}\sum _{u=1}^U\sum _{f \in {\mathcal {F}}_{3}}\sum _{k=1}^K\rho ^{2\omega }_{k}y^{2\omega }_{ufk}- {\alpha _2} \left[ \sum _{g=1}^G\sum _{u=1}^U c^{2\omega }_{gu}\left( \sum _{k=1}^K{x^{2\omega }_{guk}} \right) + \sum _{u=1}^U\sum _{f \in {\mathcal {F}}_{3}} c^{2\omega }_{uf}\left( \sum _{k=1}^K{y^{2\omega }_{ufk}} \right) \right. \right. \\&\quad \left. +\, \sum _{{f} \in {{\mathcal {F}}}_3} {c_{{f}}^{(E),2\omega }\left( \sum _{u=1}^U\sum _{k=1}^K{y^{2\omega }_{u{f}k}} \right) } + \sum _{u=1}^U {c_{u}^{(E),2\omega }\left( \sum _{g=1}^G\sum _{k=1}^K{x^{2\omega }_{guk}, \gamma _u^1,\gamma _u^{2\omega },\delta _u^{2\omega } } \right) }\right. \\&\quad \left. + \,\sum _{\tilde{f}\in \tilde{{\mathcal {F}}}_2}{c^{2\omega }_{\tilde{f}} \left( \sum _{u=1}^U\sum _{k=1}^K y^{2\omega }_{u\tilde{f}k}\right) } +\sum _{u=1}^U c_u^{2\omega }(\gamma _u^{2\omega })+\sum _{u=1}^U c_u^{(D),2\omega } (\delta _u^{2\omega })\right] \\&\quad \left. -\,{\alpha _3}\sum _{k=1}^K\beta _{k} \left[ \sum _{g=1}^G R_{gk}^{2\omega }\cdot D_{k}-\left( \sum _{u=1}^U\sum _{f\in {\mathcal {F}}_3} y_{ufk}^{2\omega }+\sum _{u=1}^{U}\sum _{f\in {\mathcal {F}}_3}y^1_{ufk}-{\sum _{g=1}^G}R^{1}_{gk}\cdot D_{k}\right) \right] \right. \\&\quad \left. +\,{\mathbb {E}}_{\Xi }[P^3(\chi ^3,\omega ,\xi )]\right\} \end{aligned} \end{aligned}$$

subject to constraints:

11 $$\begin{aligned}{} & {} \sum _{u=1}^U x^{2\omega }_{guk}{\le } R^{2\omega }_{gk}\cdot D_{k}{-\left[ \sum _{u=1}^U x^{1}_{guk}-R_{gk}^1\cdot D_{k}\right] }, \quad \forall g,\; \forall k,\;\forall \omega \in \Omega , \end{aligned}$$

12 $$\begin{aligned}{} & {} \sum _{k=1}^K\sum _{g=1}^G x^{2\omega }_{guk} \le \overline{S}_{u}+\gamma _u^{1}+\gamma _u^{2\omega }-\delta _u^{2\omega }, \quad \forall u,\; \forall \omega \in \Omega , \end{aligned}$$

13 $$\begin{aligned}{} & {} \sum _{\hat{f}\in \hat{{\mathcal {F}}}_1}y^{2\omega }_{u\hat{f}k}+\sum _{\tilde{f}\in \tilde{{\mathcal {F}}}_2}y^{2\omega }_{u\tilde{f}k} \le \sum _{g=1}^G x^{2\omega }_{guk}, \quad \forall u,\; \forall k,\; \forall \omega \in \Omega , \end{aligned}$$

14 $$\begin{aligned}{} & {} \sum _{u=1}^U\sum _{k=1}^K s_k y^{2\omega }_{ufk} \le S_{f}, \quad \forall f\in {\mathcal {F}}_{3},\; \forall \omega \in \Omega , \end{aligned}$$

15 $$\begin{aligned}{} & {} \sum _{u=1}^U y^{2\omega }_{ufk} \le M\cdot S_{fk} \quad \forall f\in {\mathcal {F}}_{3},\; \forall k=1,\ldots ,K,\; \forall \omega \in \Omega , \end{aligned}$$

16 $$\begin{aligned}{} & {} \sum _{\tilde{f}\in \tilde{{\mathcal {F}}}_{2}} {c^{1}_{\tilde{f}}\left( \sum _{u=1}^U\sum _{k=1}^K y^{1}_{u\tilde{f}k}\right) }+\sum _{u=1}^U c_u^1(\gamma _u^1)+\sum _{\tilde{f}\in \tilde{{\mathcal {F}}}_{2}} {c^{2\omega }_{\tilde{f}}\left( \sum _{u=1}^U\sum _{k=1}^K y^{2\omega }_{u\tilde{f}k}\right) }\nonumber \\{} & {} \quad +\sum _{u=1}^U c_u^{2\omega }(\gamma _u^{2\omega }) +\sum _{u=1}^U c_u^{(D),2\omega }(\delta _u^{2\omega }) \le {\overline{B}^{1}+} \overline{B}^{2\omega },\quad \forall \omega , \end{aligned}$$

17 $$\begin{aligned}{} & {} \gamma _u^{2\omega }\le \overline{\gamma }_u^{2\omega }, \quad \forall u=1,\ldots ,U,\; \forall \omega \in \Omega , \end{aligned}$$

18 $$\begin{aligned}{} & {} \delta _u^{2\omega }\le \gamma _u^{1}, \quad \forall u=1,\ldots ,U,\; \forall \omega \in \Omega , \end{aligned}$$

19 $$\begin{aligned}{} & {} x^{2\omega }_{guk},y^{2\omega }_{u\hat{f}k}, y^{2\omega }_{u\tilde{f}k}, \gamma _u^{2\omega }, \delta _u^{2\omega } \in {\mathbb {R}}_+, \forall g,\;\forall u,\;\forall \hat{f}\in \hat{{\mathcal {F}}}_1,\; \forall \tilde{f}\in \tilde{{\mathcal {F}}}_2,\; \forall k,\; \forall \omega . \end{aligned}$$

Observe that, since in stage 2 the provider can establish whether to add or to reduce the capacities of the controller UAVs, unlike the objective function of the first stage, (1), the objective function of the second stage, (10), also includes the costs due to the reduction of the capacities of controller UAVs. Therefore, the objective function (10) to maximize is given by the difference between the quantity of executed services and the total cost, to which the penalty to pay for the unmet demand is subtracted, while the expected value of the profit of the service provider in the third stage is summed. Observe that each executed service is multiplied by the priority levels (it is consistent thanks to the 5G network slicing); the total cost is multiplied by the weight $\alpha _2$, while the unmet demand, in which we denoted by $\beta _k$ the unit penalty (non-negative), is multiplied by the weight $\alpha _3$.

Constraint (11) establishes that, in stage 2 under each scenario $\omega$, the flows of transmitted data for service *k* from user or device *g* to all the controller UAVs are less than or equal to the total amount of requested data not yet satisfied (from the preparations of stage 1). Constraint (12) ensures that, under each scenario $\omega$ of the second stage, the flows of requested data for services to each controller UAV *u* are less than or equal to their maximum capacity, given by the capacity $\overline{S}_u$ to which the additional capacities of the first and second stage are summed, while the reduced capacity (of the second stage) is subtracted. Constraints (13), (14), (15), (17) and (19) have the same meaning as the constraints (4), (5), (6), (8) and (9), defined for the first stage, while (16) states that the sum of the costs due to add new UAVs or capacities in stage 1 and stage 2 summed to the cost needed to eventually reduce the capacity (in the second stage), must not exceed the sum of the budgets available for the first two stages. Furthermore, constraint (18) ensures that the reduced capacity of each controller UAV *u* (in stage 2) is less than or equal to the capacity added during the first stage.

Note that the total penalty in the objective function (10) is always nonnegative, as proved by the following Proposition.

### Proposition 3.1

The term multiplied by the unit penalty encumbered by service providers on the unmet demand for service *k*, $\beta _k$, in the objective function (10) is nonnegative, that is:

$$\begin{aligned} \left[ \sum _{g=1}^G R_{gk}^{2\omega }\cdot D_{k}-\left( \sum _{u=1}^U\sum _{f\in {\mathcal {F}}_3}y_{ufk}^{2\omega } +\sum _{u=1}^{U}\sum _{f\in {\mathcal {F}}_3}y^1_{ufk}-\sum _{g=1}^GR^{1}_{gk}\cdot D_{k}\right) \right] \ge 0,\quad \forall k,\; \forall \omega . \end{aligned}$$

### Proof

By constraint (13), observing that the sum of $y_{u\hat{f}k}^{2\omega }$ as $\hat{f}\in \hat{{\mathcal {F}}}_1$ added to the sum of $y_{u\tilde{f}k}^{2\omega }$ as $\tilde{f}\in \tilde{{\mathcal {F}}}_2$ equals the sum of $y_{ufk}^{2\omega }$ as $f\in {\mathcal {F}}_3$ $\left( \sum _{\hat{f}\in \hat{{\mathcal {F}}}_1}y_{u\hat{f}k}^{2\omega }+\sum _{\tilde{f}\in \tilde{{\mathcal {F}}}_2}y_{u\tilde{f}k}^{2\omega }=\sum _{f\in {\mathcal {F}}_3}y_{ufk}^{2\omega }\right)$, and summing to *u* (since (13) holds $\forall u=1,\cdots ,U$) we obtain:

20 $$\begin{aligned} \sum _{u=1}^U\sum _{f\in {\mathcal {F}}_3}y_{ufk}^{2\omega }\le \sum _{g=1}^G\sum _{u=1}^Ux_{guk}^{2\omega },\quad \forall k,\; \forall \omega . \end{aligned}$$

Using constraint (11), inequality (20) becomes:

$$\begin{aligned} \sum _{u=1}^U\sum _{f\in {\mathcal {F}}_3}y_{ufk}^{2\omega }\le \sum _{g=1}^G\left[ R^{2\omega }_{gk}\cdot D_{k}-\left[ \sum _{u=1}^U x^{1}_{guk}-R_{gk}^1\cdot D_{k}\right] \right] ,\quad \forall k,\; \forall \omega , \end{aligned}$$

that is:

$$\begin{aligned} \sum _{u=1}^U\sum _{f\in {\mathcal {F}}_3}y_{ufk}^{2\omega }\le \sum _{g=1}^G R^{2\omega }_{gk}\cdot D_{k}-\sum _{g=1}^G\sum _{u=1}^U x^{1}_{guk}+\sum _{g=1}^GR_{gk}^1\cdot D_{k},\quad \forall k,\; \forall \omega , \end{aligned}$$

or, equivalently:

21 $$\begin{aligned} \sum _{g=1}^G R^{2\omega }_{gk}\cdot D_{k}-\left( \sum _{u=1}^U\sum _{f\in {\mathcal {F}}_3}y_{ufk}^{2\omega }+\sum _{g=1}^G\sum _{u=1}^U x^{1}_{guk}-\sum _{g=1}^GR_{gk}^1\cdot D_{k}\right) \ge 0,\quad \forall k,\; \forall \omega . \end{aligned}$$

Moreover, remembering that constraint (4) holds for all $u=1,\ldots ,U$, and summing for *u*, we obtain:

22 $$\begin{aligned} \sum _{g=1}^G\sum _{u=1}^U x^1_{guk} \ge \sum _{u=1}^U\sum _{\hat{f}\in \hat{{\mathcal {F}}}_1}y^1_{u\hat{f}k}+\sum _{u=1}^U\sum _{\tilde{f}\in \tilde{{\mathcal {F}}}_2}y^1_{u\tilde{f}k}=\sum _{u=1}^U\sum _{f\in {\mathcal {F}}_3}y^1_{u{f}k}, \quad \forall k=1,\ldots ,K. \end{aligned}$$

From (21) and (22) we obtain:

23 $$\begin{aligned}{} & {} \sum _{g=1}^G R^{2\omega }_{gk}\cdot D_{k}-\left( \sum _{u=1}^U\sum _{f\in {\mathcal {F}}_3}y_{ufk}^{2\omega }+\sum _{u=1}^U\sum _{f\in {\mathcal {F}}_3}y^1_{u{f}k}-\sum _{g=1}^GR_{gk}^1\cdot D_{k}\right) \nonumber \\{} & {} \quad \ge \sum _{g=1}^G R^{2\omega }_{gk}\cdot D_{k}-\left( \sum _{u=1}^U\sum _{f\in {\mathcal {F}}_3}y_{ufk}^{2\omega }+\sum _{g=1}^G\sum _{u=1}^U x^{1}_{guk}-\sum _{g=1}^GR_{gk}^1\cdot D_{k}\right) \ge 0,\quad \forall k,\; \forall \omega , \end{aligned}$$

which is what we wanted to prove. $\square$

Since the weight and the unit penalty are non-negative (that is, $\alpha _{3}, \beta _{k}\ge 0,\;\forall k=1,\ldots ,K$), Proposition 3.1 guarantees that the term of the objective function (10) referring to the unmet demand is non-negative, and, then, that it is actually minimized.

We also underline that we are assuming that, while in the first stage of preparedness it was only possible to add capacity to each controller UAV *u*, under scenarios of stage 2 we can establish if it is convenient to add or reduce capacity; namely, to determine the values of $\gamma _u^{2\omega }$ and $\delta _u^{2\omega }$ variables, respectively (because stage 2 is also the preparedness phase of stage 3, in which the demand falls).

Furthermore, observe that, since in the objective function both the costs due to add capacity to each controller UAV *u*, $c_u^{2\omega }(\gamma _u^{2\omega })$, and the costs due to reduce capacity to each controller UAV *u*, $c_u^{(D),2\omega }(\delta _u^{2\omega })$, appear and, therefore, both costs are minimized, it follows that either capacity is added or reduced, but it cannot be added and reduced at the same time (during the same scenario of stage 2), because, obviously, it is not convenient.

The objective $P^3(\chi ^3,\omega ,\xi )$ is determined as the solution to the following third stage stochastic maximization problem:

24 $$\begin{aligned} \begin{aligned}&\text{ Max }\left\{ {\alpha _1}\sum _{u=1}^U\sum _{f \in {\mathcal {F}}_{3}}\sum _{k=1}^K\rho ^{3\xi }_{k}y^{3\xi }_{ufk}-{\alpha _2}\left[ \sum _{g=1}^G\sum _{u=1}^U c^{3\xi }_{gu}\left( \sum _{k=1}^K{x^{3\xi }_{guk}} \right) + \sum _{u=1}^U\sum _{f \in {\mathcal {F}}_{3}} c^{3\xi }_{uf}\left( \sum _{k=1}^K{y^{3\xi }_{ufk}} \right) \right. \right. \\&\quad \left. + \sum _{{f} \in {{\mathcal {F}}}_3} {c_{{f}}^{(E),3\xi }\left( \sum _{u=1}^U\sum _{k=1}^K{y^{3\xi }_{u{f}k}} \right) } + \sum _{u=1}^U {c_{u}^{(E),3\xi }\left( \sum _{g=1}^G\sum _{k=1}^K{x^{3\xi }_{guk}, \gamma _u^{1},\gamma _u^{2\omega },\delta _u^{2\omega }, \delta _u^{3\xi }} \right) }\right. \\&\quad \left. \left. + \sum _{\tilde{f}\in \tilde{{\mathcal {F}}}_2}{c^{3\xi }_{\tilde{f}} \left( \sum _{u=1}^U\sum _{k=1}^K y^{3\xi }_{u\tilde{f}k}\right) } +\sum _{u=1}^U c_u^{(D),3\xi }(\delta _u^{3\xi }) \right] \right\} \end{aligned} \end{aligned}$$

subject to constraints:

25 $$\begin{aligned}{} & {} \sum _{u=1}^U x^{3\xi }_{guk}{\le } R^{3\xi }_{gk}\cdot D_{k}, \quad \forall g,\; \forall k,\;\forall \xi \in \Xi , \end{aligned}$$

26 $$\begin{aligned}{} & {} \sum _{k=1}^K\sum _{g=1}^G x^{3\xi }_{guk} \le \overline{S}_{u}+\gamma _u^{1}+\gamma _u^{2\omega }-\delta _u^{2\omega } -\delta _u^{3\xi }, \quad \forall u,\; \forall \xi \in \Xi , \end{aligned}$$

27 $$\begin{aligned}{} & {} \sum _{\hat{f}\in \hat{{\mathcal {F}}}_1}y^{3\xi }_{u\hat{f}k}+\sum _{\tilde{f}\in \tilde{{\mathcal {F}}}_2}y^{3\xi }_{u\tilde{f}k} \le \sum _{g=1}^G x^{3\xi }_{guk}, \quad \forall u,\; \forall k,\; \forall \xi \in \Xi , \end{aligned}$$

28 $$\begin{aligned}{} & {} \sum _{u=1}^U\sum _{k=1}^K s_k y^{3\xi }_{ufk} \le S_{f}, \quad \forall f\in {\mathcal {F}}_{3},\; \forall \xi \in \Xi , \end{aligned}$$

29 $$\begin{aligned}{} & {} \sum _{u=1}^U y^{3\xi }_{ufk} \le M\cdot S_{fk}, \quad \forall f\in {\mathcal {F}}_{3},\; \forall k=1,\ldots , K,\; \forall \xi \in \Xi , \end{aligned}$$

30 $$\begin{aligned}{} & {} \sum _{\tilde{f}\in \tilde{{\mathcal {F}}}_{2}} {c^{1}_{\tilde{f}}\left( \sum _{u=1}^U\sum _{k=1}^K y^{1}_{u\tilde{f}k}\right) }+\sum _{u=1}^U c_u^1(\gamma _u^1)\nonumber \\{} & {} \quad +\sum _{\tilde{f}\in \tilde{{\mathcal {F}}}_{2}} {c^{2\omega }_{\tilde{f}}\left( \sum _{u=1}^U\sum _{k=1}^K y^{2\omega }_{u\tilde{f}k}\right) }+\sum _{u=1}^U c_u^{2\omega }(\gamma _u^{2\omega }) +\sum _{u=1}^U c_u^{(D),2\omega }(\delta _u^{2\omega })\nonumber \\{} & {} \quad +\sum _{\tilde{f}\in \tilde{{\mathcal {F}}}_{2}} {c^{3\xi }_{\tilde{f}} \left( \sum _{u=1}^U\sum _{k=1}^K y^{3\xi }_{u\tilde{f}k}\right) } +\sum _{u=1}^U c_u^{(D),3\xi }(\delta _u^{3\xi }) \le {\overline{B}^{1}+} \overline{B}^{2\omega },\quad \forall \xi , \end{aligned}$$

31 $$\begin{aligned}{} & {} \delta _u^{3\xi }\le \gamma _u^1+\gamma _u^{2\omega }-\delta _u^{2\omega }, \quad \forall u=1,\ldots ,U, \; \forall \omega \in \Omega _{\xi }, \; \forall \xi \in \Xi , \end{aligned}$$

32 $$\begin{aligned}{} & {} x^{3\xi }_{guk},y^{3\xi }_{u\hat{f}k}, y^{3\xi }_{u\tilde{f}k}, \delta _u^{3\xi } \in {\mathbb {R}}_+, \forall g,\;\forall u,\;\forall \hat{f}\in \hat{{\mathcal {F}}}_1,\; \forall \tilde{f}\in \tilde{{\mathcal {F}}}_2,\; \forall k,\; \forall \xi . \end{aligned}$$

With the assumption that in the third stage the provider can only remove remaining capacity from the first two stages, in this new objective function, (24), there isn’t the term related to the cost due to increasing the capacities of the controller UAVs (but, obviously, there are the costs to reduce these capacities). Moreover, constraint (25) represents the conservation law of stage 3 only: the amount of transmitted data for service *k* by user or device *g* to all the controller UAVs cannot be higher than the total amount of data demanded, $R_{gk}^{3\xi }\cdot D_{k}$. The maximum capacity in constraint (26) is given by $\overline{S}_u$ summed by the added capacities, subtracted by the reduced capacities. The budget constraint (30) takes into account the sum of the costs to use additional UAVs, to add and to reduce capacities of all the three stages and requires it is less than or equal to the sum of the available budgets of stage 1 and stage 2. We are assuming that the provider has a certain budget, $\overline{B}^1$, that it can use, in addition to special and support funding, $\overline{B}^{2\omega }$, in case of a disastrous event. Lastly, constraint (31) establishes that in stage 3 it is not possible to reduce more capacity than that added in stages 1 and 2.

Following [14, 17, 38] and the standard stochastic programming theory (see [9, 48]), the first-, second- and third-stage problems can be solved together through a unique maximization problem. Such a unique problem allows us to obtain the solutions of the complete problem (with all three stages) by solving only one optimization problem. Furthermore, this formulation guarantees the consistency of the solutions (which in the three problems are linked to each other but which must respect all the constraints at the same time). The new objective function is given by the sum of the three objective functions (1), (10) and (24), that is:

33 $$\begin{aligned}{} & {} \text{ Max }\left\{ {\alpha _1} \sum _{u=1}^U\sum _{f \in {\mathcal {F}}_{3}}\sum _{k=1}^K{\rho ^1_{k}y_{ufk}^1}-{\alpha _2}\left[ \sum _{g=1}^G\sum _{u=1}^U c^1_{gu}\left( \sum _{k=1}^K{x^1_{guk}} \right) + \sum _{u=1}^U\sum _{f \in {\mathcal {F}}_{3}} c^1_{uf}\left( \sum _{k=1}^K{y^1_{ufk}} \right) \right. \right. \nonumber \\{} & {} \quad \left. + \sum _{{f} \in {{\mathcal {F}}}_3} {c_{{f}}^{(E),1}\left( \sum _{u=1}^U\sum _{k=1}^K{y^1_{u{f}k}} \right) }+ \sum _{u=1}^U {c_{u}^{(E),1}\left( \sum _{g=1}^G\sum _{k=1}^K{x^1_{guk},\gamma _u^1 } \right) }\right. \nonumber \\{} & {} \quad \left. + \sum _{\tilde{f}\in \tilde{{\mathcal {F}}}_2}{c^1_{\tilde{f}}\left( \sum _{u=1}^U\sum _{k=1}^K y^1_{u\tilde{f}k}\right) } +\sum _{u=1}^U c_u^1(\gamma _u^1)\right] \nonumber \\{} & {} \quad +\sum _{\omega \in \Omega }\left[ p_{\omega }\left( {\alpha _1} \sum _{u=1}^U\sum _{f \in {\mathcal {F}}_{3}} \sum _{k=1}^K\rho ^{2\omega }_{k}y^{2\omega }_{ufk}-{\alpha _2}\left[ \sum _{g=1}^G \sum _{u=1}^U c^{2\omega }_{gu}\left( \sum _{k=1}^K{x^{2\omega }_{guk}} \right) + \sum _{u=1}^U\sum _{f \in {\mathcal {F}}_{3}} c^{2\omega }_{uf}\left( \sum _{k=1}^K{y^{2\omega }_{ufk}} \right) \right. \right. \right. \nonumber \\{} & {} \quad \left. + \sum _{{f} \in {{\mathcal {F}}}_3} {c_{{f}}^{(E),2\omega } \left( \sum _{u=1}^U\sum _{k=1}^K{y^{2\omega }_{u{f}k}} \right) } + \sum _{u=1}^U {c_{u}^{(E),2\omega }\left( \sum _{g=1}^G\sum _{k=1}^K{x^{2\omega }_{guk}, \gamma _u^1,\gamma _u^{2\omega },\delta _u^{2\omega } } \right) }\right. \nonumber \\{} & {} \quad \left. + \sum _{\tilde{f}\in \tilde{{\mathcal {F}}}_2}{c^{2\omega }_{\tilde{f}}\left( \sum _{u=1}^U \sum _{k=1}^K y^{2\omega }_{u\tilde{f}k}\right) } +\sum _{u=1}^U c_u^{2\omega }(\gamma _u^{2\omega }) +\sum _{u=1}^U c_u^{(D),2\omega }(\delta _u^{2\omega }) \right] \nonumber \\{} & {} -{\alpha _3}\sum _{k=1}^K\beta _{k}\left[ \sum _{g=1}^G R_{gk}^{2\omega }\cdot D_{k}-\left( \sum _{u=1}^U\sum _{f\in {\mathcal {F}}_3}y_{ufk}^{2\omega } +\sum _{u=1}^{U}\sum _{f\in {\mathcal {F}}_3}y^1_{ufk}-{\sum _{g=1}^G}R^{1}_{gk}\cdot D_{k}\right) \right] \nonumber \\{} & {} +\sum _{\xi \in \Xi }Pr(\xi |\omega )\left( {\alpha _1} \sum _{u=1}^U\sum _{f \in {\mathcal {F}}_{3}}\sum _{k=1}^K\rho ^{3\xi }_{k}y^{3\xi }_{ufk} -{\alpha _2}\left[ \sum _{g=1}^G\sum _{u=1}^U c^{3\xi }_{gu}\left( \sum _{k=1}^K{x^{3\xi }_{guk}} \right) + \sum _{u=1}^U\sum _{f \in {\mathcal {F}}_{3}} c^{3\xi }_{uf}\left( \sum _{k=1}^K{y^{3\xi }_{ufk}} \right) \right. \right. \nonumber \\{} & {} \left. + \sum _{{f} \in {{\mathcal {F}}}_3} {c_{{f}}^{(E),3\xi } \left( \sum _{u=1}^U\sum _{k=1}^K{y^{3\xi }_{u{f}k}} \right) } + \sum _{u=1}^U {c_{u}^{(E),3\xi }\left( \sum _{g=1}^G\sum _{k=1}^K{x^{3\xi }_{guk}, \gamma _u^{1},\gamma _u^{2\omega },\delta _u^{2\omega }, \delta _u^{3\xi } } \right) }\right. \nonumber \\{} & {} \left. \left. \left. \left. \left. + \sum _{\tilde{f}\in \tilde{{\mathcal {F}}}_2}{c^{3\xi }_{\tilde{f}} \left( \sum _{u=1}^U\sum _{k=1}^K y^{3\xi }_{u\tilde{f}k}\right) } +\sum _{u=1}^U c_u^{(D),3\xi }(\delta _u^{3\xi }) \right] \right) \right] \right) \right\} \end{aligned}$$

subject to constraints (2)–(9), (11)–(19) and (25)–(32).

Furthermore, let:

- $S=\{1,2,3\}$ be the set of the three stages and we denote by *s* the typical one;
- $\Theta _s$ be the set of scenarios that can occur in stage *s*, and we denote by $\theta _s$ a generic scenario that could occur. Note that:
  - If $s=1$ we have that only one scenario $\theta _1$ belongs to $\Theta _1$, the current one, and, therefore, we also obtain that $Pr(\theta _1|\theta _0)=1$ and $p_{\theta _{0}}=p_{\theta _{1}}=1$;
  - If $s=2$ we have that $\Theta _2\equiv \Omega$ and $\theta _2\in \Theta _2$ is equivalent to $\omega \in \Omega$; therefore, $Pr(\theta _2|\theta _1)=p_{\omega }$ (since each scenario $\theta _2$ originates from the only scenario $\theta _1$, it follows that $Pr(\theta _2|\theta _1)=Pr(\theta _2)=p_{\theta _{2}}$);
  - If $s=3$ we have that $\Theta _3\equiv \Xi$ and $\theta _3\in \Theta _3$ corresponds to $\xi \in \Xi$; therefore, $Pr(\theta _3|\theta _2)=Pr(\xi |\omega )$.
  Observe also that, without any loss in generality, we denote the superscript $s\theta _s$ by $s\theta$ because it is obvious that $\theta$ refers to the stage *s*. Let:
- $\gamma _u^{s'\theta '}$ and $\delta _u^{s'\theta '}$ be the vectors of additional and reduced capacities on the controller UAV *u*, respectively, composed of the quantities relating to the stages and scenarios previous or equal to *s* and $\theta _s$.
  As previously described, during the first stage we can define whether to add capacity to each controller UAV *u* ($\gamma _u^{1\theta _1}$ or, equivalently, $\gamma _u^1$), but since the first stage is the preparedness one, in this stage the capacity cannot be reduced, that is $\delta _u^{1\theta _1}=\delta _u^{1}=0,\;\forall u=1,\ldots ,U$. Analogously, during the third stage we can establish whether to reduce capacity to each controller UAV *u* ($\delta _u^{3\theta _3}$ or, equivalently, $\delta _u^{3\xi }$), while such a capacity cannot be added; namely $\gamma _u^{3\theta _3}=\gamma _u^{3\xi }=0,\;\forall u=1,\ldots ,U,\; \forall \theta _3 \in \Theta _3\; (\forall \xi \in \Xi ).$

Therefore, the unique maximization problem could be written in the following compact form:

34 $$\begin{aligned} \begin{aligned}&\text{ Max }\left\{ \sum _{s=1}^3\sum _{\begin{array}{c} \theta _s\in \Theta _s\\ \theta _{s-1}\in \Theta _{s-1} \end{array}} \left[ p_{\theta _{s-1}}Pr(\theta _s|\theta _{s-1})\left( \alpha _1\sum _{u=1}^U\sum _{f \in {\mathcal {F}}_{3}} \sum _{k=1}^K\rho ^{s\theta }_{k}y^{s\theta }_{ufk}-\alpha _2\left[ \sum _{g=1}^G \sum _{u=1}^U c^{s\theta }_{gu}\left( \sum _{k=1}^K{x^{s\theta }_{guk}} \right) \right. \right. \right. \right. \\&\quad \left. +\sum _{u=1}^U\sum _{f \in {\mathcal {F}}_{3}} c^{s\theta }_{uf}\left( \sum _{k=1}^K{y^{s\theta }_{ufk}} \right) + \sum _{{f} \in {{\mathcal {F}}}_3} {c_{{f}}^{(E),s\theta }\left( \sum _{u=1}^U\sum _{k=1}^K{y^{s\theta }_{u{f}k}} \right) } + \sum _{u=1}^U {c_{u}^{(E),s\theta }\left( \sum _{g=1}^G\sum _{k=1}^K{x^{s\theta }_{guk}, \gamma _u^{s'\theta '},\delta _u^{s'\theta '} } \right) } \right. \\&\quad \left. \left. \left. + \sum _{\tilde{f}\in \tilde{{\mathcal {F}}}_2}{c^{s\theta }_{\tilde{f}} \left( \sum _{u=1}^U\sum _{k=1}^K y^{s\theta }_{u\tilde{f}k}\right) } \right] \right) \right] -\alpha _2\left[ \sum _{s=1}^2\sum _{\begin{array}{c} \theta _s\in \Theta _s \\ \theta _{s-1}\in \Theta _{s-1} \end{array}}p_{\theta _{s-1}} Pr(\theta _s|\theta _{s-1})\left( \sum _{u=1}^U c_u^{s\theta }(\gamma _u^{s\theta }) \right) \right. \\&\quad \left. +\sum _{s=2}^3\sum _{\begin{array}{c} \theta _s\in \Theta _s \\ \theta _{s-1} \in \Theta _{s-1} \end{array}}p_{\theta _{s-1}}Pr(\theta _s|\theta _{s-1}) \left( \sum _{u=1}^U c_u^{(D),s\theta }(\delta _u^{s\theta })\right) \right] \\&\quad \left. -\sum _{\theta _2\in \Theta _2}Pr(\theta _2|\theta _1)\alpha _3\sum _{k=1}^K\beta _{k} \left[ \sum _{g=1}^G R_{gk}^{2\theta }\cdot D_{k}-\left( \sum _{u=1}^U\sum _{f\in {\mathcal {F}}_3} y_{ufk}^{2\theta }+\sum _{u=1}^{U}\sum _{f\in {\mathcal {F}}_3}y^{1\theta }_{ufk}-{\sum _{g=1}^G}R^{1\theta }_{gk}\cdot D_{k}\right) \right] \right\} \end{aligned} \end{aligned}$$

subject to constraints:

35 $$\begin{aligned}{} & {} \sum _{u=1}^U x^1_{guk}{\ge } R^1_{gk}\cdot D_{k} \quad \forall g=1,\ldots ,G,\; \forall k=1,\ldots ,K, \end{aligned}$$

36 $$\begin{aligned}{} & {} \sum _{u=1}^U x^{2\omega }_{guk}{\le } R^{2\omega }_{gk}\cdot D_{k}{-\left[ \sum _{u=1}^U x^{1}_{guk}-R_{gk}^1\cdot D_{k}\right] }, \quad \forall g,\; \forall k,\;\forall \omega \in \Omega , \end{aligned}$$

37 $$\begin{aligned}{} & {} \sum _{u=1}^U x^{3\xi }_{guk}\le R^{3\xi }_{gk}\cdot D_{k}, \quad \forall g,\; \forall k,\;\forall \xi \in \Xi , \end{aligned}$$

38 $$\begin{aligned}{} & {} \sum _{k=1}^K\sum _{g=1}^G x^{s\theta }_{guk} \le \overline{S}_{u}+\sum _{s=1}^s\sum _{\theta '\in \Theta '_\theta }(\gamma _u^{s'\theta '}-\delta _u^{s'\theta '}), \quad \forall u,\; \forall s=1,2,3,\;\forall \theta _s\in \Theta _s, \end{aligned}$$

39 $$\begin{aligned}{} & {} \sum _{\hat{f}\in \hat{{\mathcal {F}}}_1}y^{s\theta }_{u\hat{f}k}+\sum _{\tilde{f}\in \tilde{{\mathcal {F}}}_2}y^{s\theta }_{u\tilde{f}k} \le \sum _{g=1}^G x^{s\theta }_{guk}, \quad \forall u,\; \forall k,\;\forall s=1,2,3,\; \forall \theta _s\in \Theta _s, \end{aligned}$$

40 $$\begin{aligned}{} & {} \sum _{u=1}^U\sum _{k=1}^K s_k y^{s\theta }_{ufk} \le S_{f}, \quad \forall f\in {\mathcal {F}}_{3},\; \forall s=1,2,3,\;\forall \theta _s\in \Theta _s, \end{aligned}$$

41 $$\begin{aligned}{} & {} \sum _{u=1}^U y^{s\theta }_{ufk} \le M\cdot S_{fk}, \quad \forall f\in {\mathcal {F}}_{3},\;\forall k=1,\cdot , K,\; \forall s=1,2,3,\;\forall \theta _s\in \Theta _s, \end{aligned}$$

42 $$\begin{aligned}{} & {} \begin{array}{ll} &{} \sum _{s'=1}^s\sum _{\theta '\in \Theta '_\theta } \left( \sum _{\tilde{f}\in \tilde{{\mathcal {F}}}_{2}} {c^{s'\theta '}_{\tilde{f}} \left( \sum _{u=1}^U\sum _{k=1}^K y^{s'\theta '}_{u\tilde{f}k}\right) } +\sum _{u=1}^U c_u^{s'\theta '}(\gamma _u^{s'\theta '}) +\sum _{u=1}^U c_u^{(D),s'\theta '}(\delta _u^{s'\theta '})\right) \\ &{}\qquad \le \sum _{s'=1}^s\sum _{\theta '\in \Theta '_\theta }\overline{B}^{s'\theta '}\\ &{} \forall s,\;\forall \theta \in \Theta _s, \end{array} \end{aligned}$$

43 $$\begin{aligned}{} & {} \gamma _u^{s\theta }\le \overline{\gamma }_u^{s\theta }, \quad \forall u=1,\ldots ,U,\; \forall s=1,2,\;\forall \theta \in \Theta _s, \end{aligned}$$

44 $$\begin{aligned}{} & {} \delta _u^{s\theta }\le \sum _{s'=1}^{s-1}\sum _{\theta '\in \Theta _\theta ^-}(\gamma _u^{s'\theta '}-\delta _u^{s'\theta '}), \quad \forall u=1,\ldots ,U,\;\forall s=2,3,\; \forall \theta \in \Theta _s, \end{aligned}$$

45 $$\begin{aligned}{} & {} x^{s\theta }_{guk},y^{s\theta }_{u\hat{f}k}, y^{s\theta }_{u\tilde{f}k}, \gamma _u^{s\theta }, \delta _u^{s\theta } \in {\mathbb {R}}_+, \forall g,\;\forall u,\;\forall \hat{f}\in \hat{{\mathcal {F}}}_1,\; \forall \tilde{f}\in \tilde{{\mathcal {F}}}_2,\; \forall k,\; \forall s=1,2,3,\;\forall \theta \in \Theta _s. \end{aligned}$$

The objective function (34) is equivalent to (33). Constraints (35)–(37) refer to the constraints that relate the outgoing flows of transmitted data from users and devices and demanded data in stage 1, in stage 2 when scenario $\omega \in \Omega$ occurs and in stage 3 when scenario $\xi \in \Xi$ occurs, respectively; namely constraints (2), (11) and (25). Constraint (38) is the capacity constraint on each controller UAV, taking into account the added and reduced capacities. Note that $\Theta '_\theta$ in (38), (42) represents the set of scenarios previous or equal to $\theta$. Constraint (39) represents the conservation law for all the scenarios (and for all the stages). The capacity constraint is captured by constraint (40), as well as the specificity constraint (41) and the budget constraint (42). Observe that, in model (24)–(32), we assumed that no budget was added in the third stage; this could be obtained setting $\overline{B}^{3\xi }=0,\;\forall \xi \in \Xi$. However, constraint (42) has a more generic form which allows the provider to use an additional budget (if any). Constraint (43) simultaneously represents the upper-bound constraint for the additional capacities for each stage. Analogously, constraint (44) is the upper-bound constraint for the reduced capacities, where the upper-bounds are constituted by the difference between the added and the reduced capacities and $\Theta _\theta ^-$ is the set of scenarios previous to $\theta$. Finally, in the next Section, we will denote by $\Theta _{\theta _s}^+$ the set of scenarios following or equal to $\theta _s$. In this paper, without loss of generality, we assume that the same weight is given to the corresponding terms of the objective functions, regardless of the stage under consideration. Namely, the coefficients $\alpha _1$ and $\alpha _2$ are the same in the objective functions of three stages. This choice is motivated by the fact that in the real problem a greater weight is usually given to the first term of the objective function (which refers to the maximization of the services performed) than to the second (which refers to the minimization of costs), and this is true for each of the stages. Nevertheless, a generalization of this assumption is easy to deal with by considering distinct values for each stage: $\alpha _1^{s}$ and $\alpha _2^{s},\;\forall s=1,2,3$.

## Variational formulations

In this Section, we derive a variational formulation of the aforementioned constrained optimization model. Variational inequalities are a very useful tool which continues to be studied as a unifying, natural, novel, and compact framework for the formulation and solution of a wide class of unrelated problems (see [42]). Indeed, many equilibrium problems as well as optimization problems, which arise in pure and applied fields, are being solved using variational inequalities through known and new numerical methods (see, for example, [11, 13, 27, 37, 41]). The applications continue to grow, with this paper being yet another example. The advantage of determining such a formulation resides in being able, under appropriate assumptions (see [28, 36]), to easily obtain results of existence and uniqueness of the solution, given the well-developed theory. Therefore, we denote the vectors of variables as follows:

$$\begin{aligned}{} & {} X=(x_{guk}^{s\theta })_{\begin{array}{c} g=1,\ldots ,G \\ u=1,\ldots ,U \\ k=1,\ldots ,K \\ \theta \in \Theta \\ s=1,2,3 \end{array}},\;\hat{Y}=(y_{u\hat{f}k}^{s\theta })_{\begin{array}{c} u=1,\ldots ,U\\ \hat{f}\in \mathcal {\hat{F}}_1 \\ k=1,\ldots ,K\\ \theta \in \Theta \\ s=1,2,3 \end{array}},\; \tilde{Y}=(y_{u\tilde{f}k}^{s\theta })_{\begin{array}{c} u=1,\ldots ,U \\ \tilde{f}\in \mathcal {\tilde{F}}_2 \\ k=1,\ldots ,K \\ \theta \in \Theta \\ s=1,2,3 \end{array}};\\{} & {} \Gamma =(\gamma _u^{s\theta })_{\begin{array}{c} u=1,\ldots ,U \\ \theta \in \Theta , \\ s=1,2 \end{array}};\quad \Delta =(\delta _u^{s\theta })_{\begin{array}{c} u=1,\ldots ,U \\ \theta \in \Theta \\ s=2,3 \end{array}}. \end{aligned}$$

Moreover, we denote by $\displaystyle \overline{\Theta }=\sum _{s=1}^3 |\Theta _s|$, $\displaystyle \overline{\Theta }^3=\sum _{s=1}^2 |\Theta _s|$, and $\displaystyle \overline{\Theta }^1=\sum _{s=2}^3 |\Theta _s|$ the sums of the cardinalities of the sets of the scenarios of the three stages, the first two and the last two, respectively.

Making use of the classic variational inequality theory (see [36] and the references therein) and referring to other works of a similar nature (see [14, 17, 38]), we can state the following result.

### Theorem 4.1

An optimal solution to the constrained optimization problem (24), (35)–(45) can be obtained by solving the following variational inequality:

$$\begin{aligned}{} & {} {\text{ Find } (X^{*},\hat{Y}^{*},\tilde{Y}^{*},\Gamma ^{*},\Delta ^{*})\in {\mathbb {K}} \text{ s.t.: }}\nonumber \\{} & {} \quad \sum _{s=1}^3\sum _{\begin{array}{c} \theta _s\in \Theta _s \\ \theta _{s-1}\in \Theta _{s-1} \end{array}}p_{\theta _{s-1}}Pr(\theta _s|\theta _{s-1})\sum _{g=1}^G\sum _{u=1}^U\sum _{k=1}^K{\alpha _2}\left[ \frac{\partial c_{gu}^{s\theta }\left( \displaystyle \sum _{l=1}^K x_{gul}^{s\theta *}\right) }{\partial x_{guk}^{s\theta }}\right. \nonumber \\{} & {} \qquad \qquad \left. +\frac{\partial c_u^{(E),s\theta }\left( \sum _{m=1}^G\sum _{l=1}^K x_{mul}^{s\theta *},\gamma _{u}^{s'\theta '*},\delta _{u}^{s'\theta '*}\right) }{\partial x_{guk}^{s\theta }}\right] \times (x_{guk}^{s\theta }-x_{guk}^{s\theta *})\nonumber \\{} & {} \quad +\sum _{u=1}^U\sum _{\hat{f}\in {\mathcal {F}}_1}\sum _{k=1}^K\sum _{s=1}^3\sum _{\theta _s\in \Theta _s}\left[ \sum _{ \theta _{s-1}\in \Theta _{s-1}}p_{\theta _{s-1}}Pr(\theta _s|\theta _{s-1})\left( {\alpha _2}\frac{\partial c_{u\hat{f}}^{s\theta }\left( \sum _{l=1}^K y_{u\hat{f}l}^{s\theta *}\right) }{\partial y_{u\hat{f}k}^{s\theta }} \right. \right. \nonumber \\{} & {} \qquad \left. \left. +{\alpha _2}\frac{\partial c_{\hat{f}}^{(E),s\theta }\left( \sum _{m=1}^U\sum _{l=1}^K y_{m\hat{f}l}^{s\theta *}\right) }{\partial y_{u\hat{f}k}^{s\theta }}-{\alpha _1}\rho _k^{s\theta }\right) - {\Delta _{s3}\sum _{\theta _2\in \Theta _2}Pr(\theta _2|\theta _1)\alpha _3\beta _k} \right] \times (y_{u\hat{f}k}^{s\theta }-y_{u\hat{f}k}^{s\theta *})\nonumber \\{} & {} \quad +\sum _{u=1}^U\sum _{\tilde{f}\in {\mathcal {F}}_2}\sum _{k=1}^K\sum _{s=1}^3\sum _{\theta _s\in \Theta _s}\left[ \sum _{ \theta _{s-1}\in \Theta _{s-1}}p_{\theta _{s-1}}Pr(\theta _s|\theta _{s-1})\left( {\alpha _2}\frac{\partial c_{u\tilde{f}}^{s\theta }\left( \sum _{l=1}^K y_{u\tilde{f}l}^{s\theta *}\right) }{\partial y_{u\tilde{f}k}^{s\theta }}+{\alpha _2}\frac{\partial c_{\tilde{f}}^{(E),s\theta }\left( \sum _{m=1}^U\sum _{l=1}^K y_{m\tilde{f}l}^{s\theta *}\right) }{\partial y_{u\tilde{f}k}^{s\theta }}\right. \right. \nonumber \\{} & {} \qquad \left. \left. +{\alpha _2}\frac{\partial c_{\tilde{f}}^{s\theta }\left( \sum _{m=1}^U\sum _{l=1}^K y_{m\tilde{f}l}^{s\theta *}\right) }{\partial y_{u\tilde{f}k}^{s\theta }}-{\alpha _1}\rho _k^{s\theta }\right) - {\Delta _{s3}\sum _{\theta _2\in \Theta _2}Pr(\theta _2|\theta _1)\alpha _3\beta _k} \right] \times (y_{u\tilde{f}k}^{s\theta }-y_{u\tilde{f}k}^{s\theta *})\nonumber \\{} & {} \quad + \sum _{u=1}^U\sum _{s=1}^2\sum _{\theta _s\in \Theta _s}\left[ \sum _{\underline{s}=s}^3 \sum _{\begin{array}{c} \theta _{\underline{s}}\in \Theta _{\underline{s}}\cap \Theta _{\theta _s}^+ \\ \theta _{\underline{s}-1}\in \Theta _{\underline{s}-1}\cap \Theta _{\theta _{ {\underline{s}}}}' \end{array}}p_{\theta _{\underline{s}-1}}Pr(\theta _{\underline{s}}|\theta _{\underline{s}-1}){\alpha _2}\frac{\partial c_{u}^{(E),\underline{s}\theta }\left( \displaystyle \sum _{g=1}^G\sum _{k=1}^K x_{guk}^{\underline{s}\theta *},\gamma _{u}^{\underline{s}'\theta '*},\delta _u^{\underline{s}'\theta '*}\right) }{\partial \gamma _{u}^{s\theta }}\right. \nonumber \\{} & {} \qquad {\left. +\sum _{\theta _{s-1}\in \Theta _{s-1}}p_{\theta _{s-1}}Pr(\theta _s|\theta _{s-1}){\alpha _2}\frac{\partial c_u^{s\theta }(\gamma _{u}^{s\theta *})}{\partial \gamma _{u}^{s\theta }}\right] \times (\gamma _u^{s\theta }-\gamma _{u}^{s\theta *})}\\{} & {} \qquad + {\sum _{u=1}^U\sum _{s=2}^3\sum _{\theta _s\in \Theta _s}\left[ \sum _{\underline{s}=s}^3 \sum _{\begin{array}{c} \theta _{\underline{s}}\in \Theta _{\underline{s}}\cap \Theta _{\theta _s}^+ \\ \theta _{\underline{s}-1}\in \Theta _{\underline{s}-1}\cap \Theta _{\theta _{ {\underline{s}}}}' \end{array}}p_{\theta _{\underline{s}-1}}Pr(\theta _{\underline{s}}|\theta _{\underline{s}-1}){\alpha _2}\frac{\partial c_{u}^{(E),\underline{s}\theta }\left( \displaystyle \sum _{g=1}^G\sum _{k=1}^K x_{guk}^{\underline{s}\theta *},\gamma _{u}^{\underline{s}'\theta '*},\delta _u^{\underline{s}'\theta '*}\right) }{\partial \delta _{u}^{s\theta }}\right. }\nonumber \\{} & {} \qquad {\left. +\sum _{\theta _{s-1}\in \Theta _{s-1}}p_{\theta _{s-1}}Pr(\theta _s|\theta _{s-1}){\alpha _2}\frac{\partial c_u^{(D),s\theta }(\delta _{u}^{s\theta *})}{\partial \delta _{u}^{s\theta }}\right] \times (\delta _u^{s\theta }-\delta _{u}^{s\theta *})\ge 0} \end{aligned}$$

46 $$\begin{aligned} \forall (X,\hat{Y},\tilde{Y},\Gamma ,\Delta )\in {\mathbb {K}}, \end{aligned}$$

where $\Delta _{s3}=1$ if $s=1$ or $s=2$, null otherwise and

$$\begin{aligned} {\begin{aligned} {\mathbb {K}}:=&\Big \{(X,\hat{Y},\tilde{Y},\Gamma ,\Delta )\in \mathbb {R}_+^{U[K\overline{\Theta }(G+\hat{F}_1 +\tilde{F}_2)+\overline{\Theta }^3+\overline{\Theta }^1]}: \quad (35)-(45) \text{ hold } \Big \}. \end{aligned}} \end{aligned}$$

We now put variational inequality (46) into standard form (see [36]), that is: determine ${\mathcal {X}}^*\in {\mathcal {K}}$ such that:

47 $$\begin{aligned} \langle F({{\mathcal {X}}}),{{\mathcal {X}}}-{{\mathcal {X}}}^*\rangle \ge 0,\quad \forall {{\mathcal {X}}}\in {\mathcal {K}}, \end{aligned}$$

where ${\mathcal {K}}$ is a closed and convex set. In order to do this, we put

$$\begin{aligned} {{\mathcal {X}}}\equiv (X,\hat{Y},\tilde{Y},\Gamma ,\Delta ), \end{aligned}$$

$$F({{\mathcal {X}}})\equiv (F_i({{\mathcal {X}}}))_{i=1,\dots ,5}$$ and $${\mathcal {K}}\equiv {\mathbb {K}} $$, where:

$$\begin{aligned} \begin{aligned} F_{guk{,1}}^{s\theta }=&{\sum _{\theta _{s-1}\in \Theta _{s-1}}p_{\theta _{s-1}}Pr(\theta _s|\theta _{s-1})\alpha _2}\left[ \frac{\partial c_{gu}^{s\theta }\left( \displaystyle \sum _{l=1}^K x_{gul}^{s\theta }\right) }{\partial x_{guk}^{s\theta }} +\frac{\partial c_u^{(E),s\theta }\left( \sum _{m=1}^G\sum _{l=1}^K x_{mul}^{s\theta },\gamma _{u}^{s'\theta '},\delta _{u}^{s'\theta '}\right) }{\partial x_{guk}^{s\theta }}\right] ,\quad \forall g,u,k,s,\theta ,\\ F_{u\hat{f}k{,2}}^{s\theta }=&\left[ {\sum _{ \theta _{s-1}\in \Theta _{s-1}}}p_{\theta _{s-1}} Pr(\theta _s|\theta _{s-1})\left( {\alpha _2}\frac{\partial c_{u\hat{f}}^{s\theta } \left( \sum _{l=1}^K y_{u\hat{f}l}^{s\theta }\right) }{\partial y_{u\hat{f}k}^{s\theta }}+{\alpha _2}\frac{\partial c_{\hat{f}}^{(E),s\theta } \left( \sum _{m=1}^U\sum _{l=1}^K y_{m\hat{f}l}^{s\theta }\right) }{\partial y_{u\hat{f}k}^{s\theta }}-{\alpha _1}\rho _k^{s\theta }\right) \right. \\&\quad \left. -{\Delta _{s3}\sum _{\theta _2\in \Theta _2}Pr(\theta _2|\theta _1)\alpha _3\beta _k} \right] ,\quad \forall u,\hat{f},k,s,\theta ,\\ F_{u\tilde{f}k{,3}}^{s\theta }=&\left[ {\sum _{ \theta _{s-1}\in \Theta _{s-1}}}p_{\theta _{s-1}} Pr(\theta _s|\theta _{s-1})\left( {\alpha _2}\frac{\partial c_{u\tilde{f}}^{s\theta } \left( \sum _{l=1}^K y_{u\tilde{f}l}^{s\theta }\right) }{\partial y_{u\tilde{f}k}^{s\theta }} +{\alpha _2}\frac{\partial c_{\tilde{f}}^{(E),s\theta }\left( \sum _{m=1}^U \sum _{l=1}^K y_{m\tilde{f}l}^{s\theta }\right) }{\partial y_{u\tilde{f}k}^{s\theta }}\right. \right. \\&\quad \left. \left. +{\alpha _2}\frac{\partial c_{\tilde{f}}^{s\theta } \left( \sum _{m=1}^U\sum _{l=1}^K y_{m\tilde{f}l}^{s\theta }\right) }{\partial y_{u\tilde{f}k}^{s\theta }} -{\alpha _1}\rho _k^{s\theta }\right) - {\Delta _{s3}\sum _{\theta _2\in \Theta _2}Pr(\theta _2|\theta _1)\alpha _3\beta _k} \right] ,\quad \forall u,\tilde{f},k,s,\theta ,\\ F_{u{,4}}^{s\theta }=&\left[ \sum _{\underline{s}=s}^3\sum _{\begin{array}{c} \theta _{\underline{s}}\in \Theta _{\underline{s}} \cap \Theta _{\theta _s}^+ \\ \theta _{\underline{s}-1}\in \Theta _{\underline{s}-1}\cap \Theta _{\theta _{ {\underline{s}}}}' \end{array}}p_{\theta _{\underline{s}-1}}Pr(\theta _{\underline{s}}|\theta _{\underline{s}-1}){\alpha _2} \frac{\partial c_{u}^{(E),\underline{s}\theta }\left( \displaystyle \sum _{g=1}^G\sum _{k=1}^K x_{guk}^{\underline{s}\theta }, \gamma _{u}^{\underline{s}'\theta '},\delta _u^{\underline{s}'\theta '}\right) }{\partial \gamma _{u}^{s\theta }}\right. \\&\qquad \left. +{\sum _{ \theta _{s-1}\in \Theta _{s-1}}}p_{\theta _{s-1}}Pr(\theta _s|\theta _{s-1}){\alpha _2} \frac{\partial c_u^{s\theta }(\gamma _{u}^{s\theta })}{\partial \gamma _{u}^{s\theta }}\right] ,\quad \forall u,s,\theta , \end{aligned} \end{aligned}$$

and

$$\begin{aligned} \begin{aligned} F_{u{,5}}^{s\theta }=&\left[ \sum _{\underline{s}=s}^3\sum _{\begin{array}{c} \theta _{\underline{s}}\in \Theta _{\underline{s}} \cap \Theta _{\theta _s}^+ \\ \theta _{\underline{s}-1}\in \Theta _{\underline{s}-1}\cap \Theta _{\theta _{ {\underline{s}}}}' \end{array}}p_{\theta _{\underline{s}-1}}Pr(\theta _{\underline{s}}|\theta _{\underline{s}-1}){\alpha _2} \frac{\partial c_{u}^{(E),\underline{s}\theta }\left( \displaystyle \sum _{g=1}^G\sum _{k=1}^K x_{guk}^{\underline{s}\theta }, \gamma _{u}^{\underline{s}'\theta '},\delta _u^{\underline{s}'\theta '}\right) }{\partial \delta _{u}^{s\theta }}\right. \\&\quad \left. +{\sum _{\theta _{s-1}\in \Theta _{s-1}}}p_{\theta _{s-1}}Pr(\theta _s|\theta _{s-1}){\alpha _2} \frac{\partial c_u^{(D),s\theta }(\delta _{u}^{s\theta })}{\partial \delta _{u}^{s\theta }}\right] ,\quad \forall u,s,\theta . \end{aligned} \end{aligned}$$

Under the imposed assumptions, the function $F({{\mathcal {X}}})$ that enters variational inequality (47) is continuous. Now we prove that the feasible set ${\mathcal {K}}$ is compact. Thereby, the existence of a solution to variational inequality (47) will be guaranteed from the classical variational inequality theory (see [28]).

### Proposition 4.2

The feasible set ${\mathcal {K}}$ defined above is compact.

### Proof

From the nature of constraints (35)–(45), we can easily deduce that the set is closed. We prove that the feasible set ${\mathcal {K}}$ is also bounded. First we observe that each group of variables is lower-bounded due to constraint (45). Constraint (44) can be rewritten as follows:

$$\begin{aligned} \delta _{su}^{s\theta }+\sum _{s'=1}^{s-1}\sum _{\theta '\in \Theta _{\theta }^{-}}\delta _{u}^{s'\theta '}\le \sum _{s'=1}^{s-1}\sum _{\theta '\in \Theta _{\theta }^{-}}\gamma _u^{s'\theta '} \end{aligned}$$

and, from constraint (43), we have:

$$\begin{aligned} \delta _{u}^{s\theta }+\sum _{s'=1}^{s-1}\sum _{\theta '\in \Theta _{\theta }^{-}}\delta _{u}^{s'\theta '}\le \sum _{s'=1}^{s-1}\sum _{\theta '\in \Theta _{\theta }^{-}}\gamma _u^{s'\theta '}\le \sum _{s'=1}^{s-1}\sum _{\theta '\in \Theta _{\theta }^{-}}\overline{\gamma }_u^{s'\theta '}. \end{aligned}$$

Therefore, the group of variables $\delta _{u}^{s\theta }$, for all $u,s,\theta$, are upper-bounded. From the limitation of these variables and from constraint (38), we have that also all the variables $x_{guk}^{s\theta }$, for all $g,u,k,s,\theta$, are upper-bounded. Finally, constraints (39) and (40) ensure the limitation of the variables $y_{ufk}^{s\theta }$, for all $u,f,k,s,\theta$.

Hence, the feasible set ${\mathcal {K}}$ is compact, since it is both closed and bounded. $\square$

Given the nonlinearity of the budget constraints (42) and the related computational difficulties that could cause, we now deduce an alternative variational formulation of the proposed optimization model that allows us to relax such constraints using the associated Lagrange multipliers. We have the following:

### Theorem 4.3

A vector ${{\mathcal {X}}}^*\in {\mathcal {K}}$ is a solution to variational inequality (47) if and only if there exists a vector of Lagrange multipliers $$\lambda ^*=(\lambda ^{s\theta *})_{\begin{array}{c} s=1,2,3\\ \theta \in \Theta _{{s}} \end{array}}\in {\mathbb {R}}_+^{\overline{\Theta } }$$ such that the vector $({{\mathcal {X}}}^*,\lambda ^*)\in {\mathcal {K}}_2$ is a solution to variational inequality:

Determine $({{\mathcal {X}}}^*,\lambda ^*)\in {\mathcal {K}}_2$ such that:

$$\begin{aligned}{} & {} \sum _{s=1}^3\sum _{\begin{array}{c} \theta _s\in \Theta _s \\ \theta _{s-1}\in \Theta _{s-1} \end{array}}p_{\theta _{s-1}}Pr(\theta _s|\theta _{s-1})\sum _{g=1}^G\sum _{u=1}^U\sum _{k=1}^K{\alpha _2}\left[ \frac{\partial c_{gu}^{s\theta }\left( \displaystyle \sum _{l=1}^K x_{gul}^{s\theta *}\right) }{\partial x_{guk}^{s\theta }}\right. \\{} & {} \qquad \left. +\frac{\partial c_u^{(E),s\theta }\left( \sum _{m=1}^G\sum _{l=1}^K x_{mul}^{s\theta *},\gamma _{u}^{s'\theta '*},\delta _{u}^{s'\theta '*}\right) }{\partial x_{guk}^{s\theta }}\right] \times (x_{guk}^{s\theta }-x_{guk}^{s\theta *})\\{} & {} \quad +\sum _{u=1}^U\sum _{\hat{f}\in {\mathcal {F}}_1}\sum _{k=1}^K\sum _{s=1}^3\sum _{\theta _s\in \Theta _s}\left[ \sum _{ \theta _{s-1}\in \Theta _{s-1}}p_{\theta _{s-1}}Pr(\theta _s|\theta _{s-1})\left( {\alpha _2}\frac{\partial c_{u\hat{f}}^{s\theta }\left( \sum _{l=1}^K y_{u\hat{f}l}^{s\theta *}\right) }{\partial y_{u\hat{f}k}^{s\theta }} \right. \right. \\{} & {} \qquad \left. \left. +{\alpha _2}\frac{\partial c_{\hat{f}}^{(E),s\theta } \left( \sum _{m=1}^U\sum _{l=1}^K y_{m\hat{f}l}^{s\theta *}\right) }{\partial y_{u\hat{f}k}^{s\theta }}-{\alpha _1}\rho _k^{s\theta }\right) - {\Delta _{s3}\sum _{\theta _2\in \Theta _2}Pr(\theta _2|\theta _1)\alpha _3\beta _k} \right] \times (y_{u\hat{f}k}^{s\theta }-y_{u\hat{f}k}^{s\theta *})\\{} & {} \quad +\sum _{u=1}^U\sum _{\tilde{f}\in {\mathcal {F}}_2}\sum _{k=1}^K\sum _{s=1}^3\sum _{\theta _s\in \Theta _s} \left[ \sum _{ \theta _{s-1}\in \Theta _{s-1}}p_{\theta _{s-1}}Pr(\theta _s|\theta _{s-1}) \left( {\alpha _2}\frac{\partial c_{u\tilde{f}}^{s\theta } \left( \sum _{l=1}^K y_{u\tilde{f}l}^{s\theta *}\right) }{\partial y_{u\tilde{f}k}^{s\theta }} +{\alpha _2}\frac{\partial c_{\tilde{f}}^{(E),s\theta }\left( \sum _{m=1}^U \sum _{l=1}^K y_{m\tilde{f}l}^{s\theta *}\right) }{\partial y_{u\tilde{f}k}^{s\theta }}\right. \right. \\{} & {} \qquad \left. +{\alpha _2}\frac{\partial c_{\tilde{f}}^{s\theta }\left( \sum _{m=1}^U\sum _{l=1}^K y_{m\tilde{f}l}^{s\theta *}\right) }{\partial y_{u\tilde{f}k}^{s\theta }} -{\alpha _1}\rho _k^{s\theta }\right) - {\Delta _{s3}\sum _{\theta _2\in \Theta _2}Pr(\theta _2|\theta _1)\alpha _3\beta _k} \\{} & {} \qquad \left. +\sum _{s'={s}}^{{3}}\sum _{\theta '\in {\Theta _{\theta _s}^+}}{\lambda ^{s'\theta '*}} \frac{\partial c_{\tilde{f}}^{{s\theta }}\left( \displaystyle \sum _{m=1}^U \sum _{l=1}^Ky_{m\tilde{f}l}^{{s\theta *}}\right) }{\partial y_{u\tilde{f}k}^{s\theta }}\right] \times (y_{u\tilde{f}k}^{s\theta }-y_{u\tilde{f}k}^{s\theta *})\\{} & {} \quad +\sum _{u=1}^U\sum _{s=1}^2\sum _{\theta _s\in \Theta _s}\left[ \sum _{\underline{s}=s}^3 \sum _{\begin{array}{c} \theta _{\underline{s}}\in \Theta _{\underline{s}}\cap \Theta _{\theta _s}^+ \\ \theta _{\underline{s}-1}\in \Theta _{\underline{s}-1}\cap \Theta _{\theta _{ \underline{s}}}' \end{array}}p_{\theta _{\underline{s}-1}}Pr(\theta _{\underline{s}}|\theta _{\underline{s}-1}) \alpha _2\frac{\partial c_{u}^{(E),\underline{s}\theta }\left( \displaystyle \sum _{g=1}^G \sum _{k=1}^K x_{guk}^{\underline{s}\theta *},\gamma _{u}^{\underline{s}'\theta '*}, \delta _u^{\underline{s}'\theta '*}\right) }{\partial \gamma _{u}^{s\theta }}\right. \\{} & {} \qquad \left. + \sum _{\theta _{s-1}\in \Theta _{s-1}}Pr(\theta _s|\theta _{s-1})\alpha _2\frac{\partial c_u^{s\theta } (\gamma _{u}^{s\theta *})}{\partial \gamma _{u}^{s\theta }} +\sum _{s'=s}^3\sum _{\theta '\in \Theta _{\theta _s}^+}\lambda ^{s'\theta '*}\frac{\partial c_{u}^{s\theta } \left( \displaystyle \gamma _u^{s\theta *}\right) }{\partial \gamma _u^{s\theta }}\right] \times (\gamma _u^{s\theta } -\gamma _{u}^{s\theta *})\\{} & {} \quad +\sum _{u=1}^U\sum _{s=2}^3\sum _{\theta _s\in \Theta _s}\left[ \sum _{\underline{s}=s}^3\sum _{\begin{array}{c} \theta _{\underline{s}}\in \Theta _{\underline{s}}\cap \Theta _{\theta _s}^ + \\ \theta _{\underline{s}-1}\in \Theta _{\underline{s}-1}\cap \Theta _{\theta _{ \underline{s}}}' \end{array}}p_{\theta _{\underline{s}-1}}Pr(\theta _{\underline{s}}|\theta _{\underline{s}-1}) \alpha _2\frac{\partial c_{u}^{(E),\underline{s}\theta }\left( \displaystyle \sum _{g=1}^G \sum _{k=1}^K x_{guk}^{\underline{s}\theta *},\gamma _{u}^{\underline{s}'\theta '*}, \delta _u^{\underline{s}'\theta '*}\right) }{\partial \delta _{u}^{s\theta }}\right. \\{} & {} \qquad \left. +\sum _{\theta _{s-1}\in \Theta _{s-1}}p_{\theta _{s-1}}Pr(\theta _s|\theta _{s-1})\alpha _2 \frac{\partial c_u^{(D),s\theta }(\delta _{u}^{s\theta *})}{\partial \delta _{u}^{s\theta }} +\sum _{s'=s}^3\sum _{\theta '\in \Theta _{\theta _s}^+}\lambda ^{s'\theta '*}\frac{\partial c_{u}^{(D),s\theta } \left( \displaystyle \delta {u}^{s\theta *}\right) }{\partial \delta _u^{s\theta }}\right] \times (\delta _u^{s\theta }-\delta _{u}^{s\theta *})\\{} & {} +\sum _{s=1}^3\sum _{\theta \in \Theta _{s}}\left[ \sum _{s'=1}^s\sum _{\theta '\in \Theta '_\theta } \overline{B}^{s'\theta '}-\sum _{s'=1}^s\sum _{\theta '\in \Theta '_\theta }\left( \sum _{\tilde{f}\in \tilde{{\mathcal {F}}}_{2}} {c^{s'\theta '}_{\tilde{f}}\left( \sum _{u=1}^U \sum _{k=1}^K y^{s'\theta '*}_{u\tilde{f}k}\right) }\right. \right. \\{} & {} \qquad \left. \left. +\sum _{u=1}^U c_u^{s'\theta '}(\gamma _u^{s'\theta '*}) +\sum _{u=1}^U c_u^{(D),s'\theta '}(\delta _u^{s'\theta '*})\right) \right] \times (\lambda ^{s\theta }-\lambda ^{s\theta *})\ge 0 \end{aligned}$$

48 $$\begin{aligned} \forall ({{\mathcal {X}}},\lambda )\in {\mathcal {K}}_2 \end{aligned}$$

where $${\mathcal {K}}_2={\mathcal {K}}\times \mathbb {R}_+^{\overline{\Theta } }$$.

## Illustrative numerical example

In this Section, we provide a detailed numerical example to illustrate some key aspects of the model and to validate its effectiveness.

**Fig. 3:**
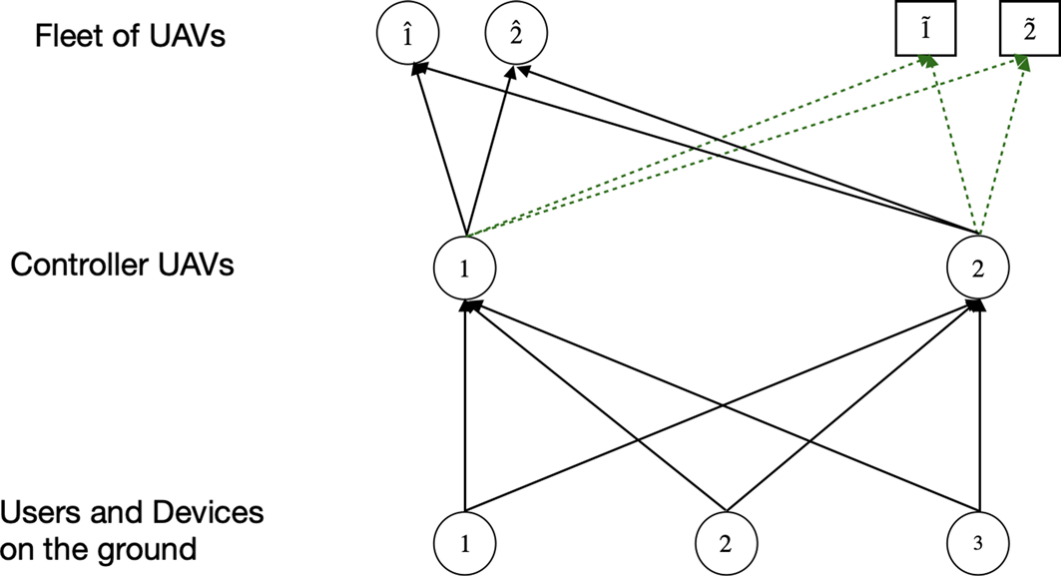
Network topology for the numerical example

For the numerical implementation, we consider the network depicted in Fig. 3. Therefore, we have $G=3$ users or devices on the ground, whose requests are received by $U=2$ controller UAVs at the middle level of the network. These requests are sent, in turn, to the fleet of UAVs at the the highest level of the network, where they perform the services requested and which consist of $\hat{{\mathcal {F}}}_1=2$ pre-existing UAVs and $\tilde{{\mathcal {F}}}_2=2$ additional UAVs. The size of this instance is constructed for easy interpretation purposes. Furthermore, we assume that large geographical areas are divided into smaller zones that can be covered by a limited number of UAVs. It should be noted that, in many practical situations, the networks are comprised of a very large number of users and devices requiring services and a large number of UAVs. This is an important issue that arises when determining the optimal management of such networks. In fact, due to the computational complexity, implementation by a single centralized unit may be too burdensome, since such a centralization would entail evident problems in terms of efficiency of the communication activities. In these situations, the main idea consists in using the distributed architectures, which consist of several components that can cooperate with one another over a unique communication network in order to achieve a specific objective or goal. Each component of a distributed architecture is associated with a group of users and devices or UAVs and is capable of managing the flows of only the users and devices or UAVs belonging to that group. Furthermore, in addition to the use of distributed architectures, to manage large areas, we suggest partitioning these zones into smaller areas which could be managed more easily.

We focus on the service about the acquisition and collection of real-time data from sensors ($K=1$). Note that, in the normalcy scenario *I*, the demands for the service have low values, since no disaster event has occurred yet, and data are acquired only for the monitoring of and the prediction of future disasters (analogously for the third stage). At stage 2, if no disaster event occurs (we are in $\omega _1 \equiv I$ scenario), the demands for the service remain unaltered with respect to stage 1; if a landslide occurs ($\omega _2$), the demands for real-time data acquisition and collection have generally higher values (but we also assumed that the demand value vanishes for a certain geographical region); if an earthquake occurs ($\omega _3$), the demands for the service are much greater than the other cases since it represents a more serious (and extended) scenario. We assume that, at stage 3, the normalcy situation can be restored, intermediate situations may occur (such as aftershocks of different severity) or the disastrous event can continue. Obviously, the model proposed in this paper can be applied to services and disastrous events different than those considered here.

In this example, we suppose that, at stage 2, three possible scenarios, $\omega _1 \equiv I,\ \omega _2$ and $\omega _3$, can occur with different probabilities:

$$\begin{aligned} p_{\omega _1}=0.2,\ p_{\omega _2}=0.5,\ p_{\omega _3}=0.3. \end{aligned}$$

**Fig. 4:**
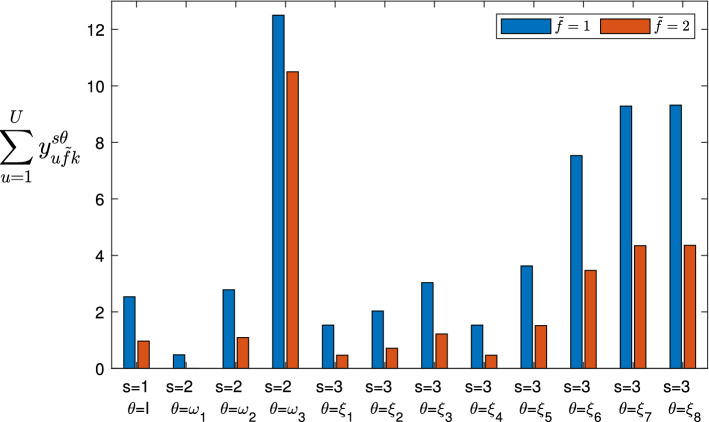
Optimal solutions: additional UAVs

We denoted by *I* the initial scenario, that is, a scenario in which no disaster event has occurred or a scenario in which it was possible to restore the starting condition of stage 1; that is, when a landslide occurs, only a portion of added capacities are reduced (because some of them are used also during the following scenarios in stage 3) and during the third scenario of stage 2, that is when an earthquake occurs, the maximum allowed capacity of the first controller UAV and much of the additional capacity of the second are used (see Fig. 4) because we assumed that the earthquake is the most serious event.

**Fig. 5:**
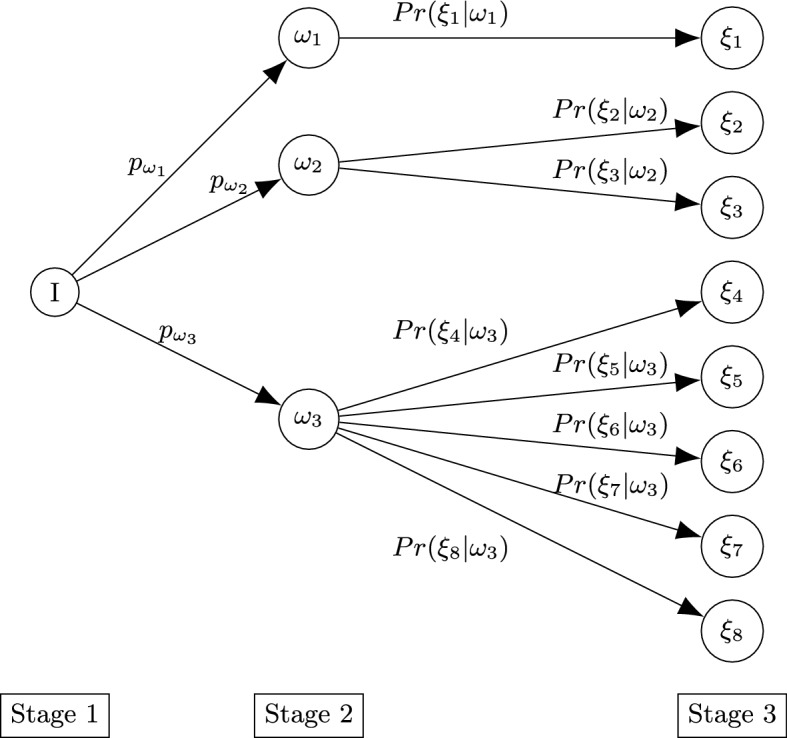
Scenarios of the three stages

Moreover, we assume that (see Fig. 5 for a more detailed depiction of the correct succession of the various scenarios between the various stages):

- From scenario $\omega _1\equiv I$ of the second stage, only the same scenario $\xi _1\equiv I$ can occur at stage 3, with a conditional probability $Pr(\xi _1|\omega _1)=1$;
- From scenario $\omega _2$ of the second stage, only two scenarios can occur at stage 3, $\xi _2$ and $\xi _3$, with conditional probabilities $Pr(\xi _1|\omega _2)=0.6$ and $Pr(\xi _2|\omega _2)=0.4$;
- From scenario $\omega _3$ of the second stage, 5 different scenarios can occur: $\xi _4\equiv I$, $\xi _5$, $\xi _6$, $\xi _7$ and $\xi _8\equiv \omega _3$, with conditional probabilities $Pr(\xi _4|\omega _3)=0.1$, $Pr(\xi _5|\omega _3)=0.35$, $Pr(\xi _6|\omega _3)=0.25$, $Pr(\xi _7|\omega _3)=0.2$ and $Pr(\xi _8|\omega _3)=0.1$.

Observe that the sum of probabilities of scenarios which can occur from each scenario is equal to 1. Moreover, since a scenario of the third stage can occur only if the previous scenario in stage 2 has occurred, it is clear that the occurrence probability of a scenario of the third stage is given by the product between the probability of the scenario from which it comes and its conditional probability: $p_{\xi _i}=Pr(\xi _i | \omega _j)p_{\omega _j}$. Therefore, the sum of probabilities of all the scenarios of stage 3 also equals 1.

Furthermore, both at the second and at the third stage, we have assumed, without loss of generality, that the order of gravity of the scenarios is in ascending order. Therefore, for instance, in stage 2, scenario $\omega _3$ is more serious in terms of disaster than the scenario $\omega _2$. In the same way, at stage 3, scenario $\xi _3$, following from scenario $\omega _2$ of stage 2, is more serious than $\xi _2$, that follows from the same scenario $\omega _2$ of stage 2. Finally, scenario $\xi _8$, which is the most serious of the scenarios that follow from the scenario $\omega _3$ at stage 2, represents the same scenario $\omega _3$ in terms of severity. Therefore, scenarios $\xi _5,\ \xi _6$ and $\xi _7$ have an intermediate severity between the initial scenario $\xi _4\equiv I$ and the disaster scenario $\xi _8\equiv \omega _3$.

For the numerical setting, we select the parameters as follows:

$$\begin{aligned}{} & {} D_1=1,\ \overline{S}_1=5,\ \overline{S}_2=7,\ S_{\hat{1}}=10,\ S_{\hat{2}}=12,\ S_{\tilde{1}}=25,\ S_{\tilde{2}}=30,\ s_{1}=2;\\{} & {} S_{\hat{1}1}= S_{\hat{2}1}= S_{\tilde{1}1}= S_{\tilde{2}1}=1,\ \alpha _1=10,\ \alpha _2=1,\ \alpha _3=1; \end{aligned}$$

*Stage 1:*

$$\begin{aligned} R_{11}^1=1,\ R_{21}^1=2,\ R_{31}^1=3,\ \rho ^1_1=1,\ \overline{B}^1=100,\ \overline{\gamma }_1^1=3,\ \overline{\gamma }_2^1= 3; \end{aligned}$$

*Stage 2, scenario* $$\omega _1\equiv I$$:

$$\begin{aligned} R_{11}^{2\omega _1}=1,\ R_{21}^{2\omega _1}=2, R_{31}^{2\omega _1}=3, \ \rho _1^{2\omega _1}=1,\ \overline{B}^{2\omega _1}=0,\ \overline{\gamma }_1^{2\omega _1}=10,\ \overline{\gamma }_2^{2\omega _1}=10; \end{aligned}$$

*Stage 2, scenario* $$\omega _2$$:

$$\begin{aligned} R_{11}^{2\omega _2}=5,\ R_{21}^{2\omega _2}=0, R_{31}^{2\omega _2}=10, \ \rho _1^{2\omega _2}=1,\ \overline{B}^{2\omega _2}=500,\ \overline{\gamma }_1^{2\omega _2}=10,\ \overline{\gamma }_2^{2\omega _2}=10; \end{aligned}$$

*Stage 2, scenario* $$\omega _3$$:

$$\begin{aligned} R_{11}^{2\omega _3}=13,\ R_{21}^{2\omega _3}=10, R_{31}^{2\omega _3}=15, \ \rho _1^{2\omega _3}=1,\ \overline{B}^{2\omega _3}=1000,\ \overline{\gamma }_1^{2\omega _3}=10,\ \overline{\gamma }_2^{2\omega _3}=10; \end{aligned}$$

*Stage 3, scenario* $\xi _1\equiv I$ *from scenario* $\omega _1$:

$$\begin{aligned} R_{11}^{3\xi _1}=1,\ R_{21}^{3\xi _1}=2, R_{31}^{3\xi _1}=3, \ \rho _1^{3\xi _1}=1;\ \end{aligned}$$

*Stage 3, scenario* $\xi _2$ *from scenario* $\omega _2$:

$$\begin{aligned} R_{11}^{3\xi _2}=2,\ R_{21}^{3\xi _2}=1, R_{31}^{3\xi _2}=5, \ \rho _1^{3\xi _2}=1; \end{aligned}$$

*Stage 3, scenario* $\xi _3$ *from scenario* $\omega _2$:

$$\begin{aligned} R_{11}^{3\xi _3}=4,\ R_{21}^{3\xi _3}=1, R_{31}^{3\xi _3}=7, \ \rho _1^{3\xi _3}=1; \end{aligned}$$

*Stage 3, scenario* $\xi _4\equiv I$ *from scenario* $\omega _3$:

$$\begin{aligned} R_{11}^{3\xi _4}=1,\ R_{21}^{3\xi _4}=2, R_{31}^{3\xi _4}=3, \ \rho _1^{3\xi _4}=1; \end{aligned}$$

*Stage 3, scenario* $\xi _5$ *from scenario* $\omega _3$:

$$\begin{aligned} R_{11}^{3\xi _5}=4,\ R_{21}^{3\xi _5}=4, R_{31}^{3\xi _5}=6, \ \rho _1^{3\xi _5}=1; \end{aligned}$$

*Stage 3, scenario* $\xi _6$ *from scenario* $\omega _3$:

$$\begin{aligned} R_{11}^{3\xi _6}=7,\ R_{21}^{3\xi _6}=6, R_{31}^{3\xi _6}=9, \ \rho _1^{3\xi _6}=1; \end{aligned}$$

*Stage 3, scenario* $\xi _7$ *from scenario* $\omega _3$:

$$\begin{aligned} R_{11}^{3\xi _7}=10,\ R_{21}^{3\xi _7}=8, R_{31}^{3\xi _7}=12, \ \rho _1^{3\xi _7}=1; \end{aligned}$$

*Stage 3, scenario* $\xi _8\equiv \omega _3$ *from scenario* $\omega _3$:

$$\begin{aligned} R_{11}^{3\xi _8}=13,\ R_{21}^{3\xi _8}=10, R_{31}^{3\xi _8}=15, \ \rho _1^{3\xi _8}=1. \end{aligned}$$

Moreover, we suppose the following general expressions for the cost functions, for all $g,u,\hat{f},\tilde{f}$ and $s,\ \theta$:

$$\begin{aligned}{} & {} c_{gu}^{s\theta }=a_{gu}\left( \sum _{k=1}^Kx_{guk}^{s\theta }\right) ^2 +\overline{a}_{gu}\left( \sum _{k=1}^Kx_{guk}^{s\theta }\right) ,\\{} & {} c_{uf}^{s\theta }=d_{uf}\left( \sum _{k=1}^Kx_{ufk}^{s\theta }\right) ^2 +\overline{d}_{uf}\left( \sum _{k=1}^Kx_{ufk}^{s\theta }\right) ,\\{} & {} c_{f}^{s\theta ,(E)}=g_f\left( \sum _{u=1}^U\sum _{k=1}^K{x_{ufk}^{s\theta }} \right) ^2 +\overline{g}_f\left( \sum _{u=1}^U\sum _{k=1}^Kx_{ufk}^{s\theta }\right) ,\\{} & {} c_{u}^{(E),1}\left( \sum _{g=1}^G\sum _{k=1}^K x_{guk}^{1}, \gamma _u^1\right) =i_{u}\left( \sum _{g=1}^G\sum _{k=1}^K x_{guk}^{1}\right) ^2+\overline{i}_u\left( \sum _{g=1}^G \sum _{k=1}^K x_{guk}^{1}\right) \\{} & {} \qquad \qquad \qquad \qquad \qquad \qquad \qquad \qquad +j_u(\gamma _u^1)^2+\overline{j}_{u}(\gamma _u^1),\\{} & {} c_{u}^{(E),2\omega }\left( \sum _{g=1}^G\sum _{k=1}^K x_{guk}^{2\omega },\gamma _u^1, \gamma _u^{2\omega },\delta _u^{2\omega }\right) =i_{u}\left( \sum _{g=1}^G\sum _{k=1}^K x_{guk}^{1}\right) ^2\\{} & {} \quad +\overline{i}_u\left( \sum _{g=1}^G\sum _{k=1}^K x_{guk}^{1}\right) +j_u(\gamma _u^1)^2+\overline{j}_{u}(\gamma _u^1)\\{} & {} +k_u(\gamma _{u}^{2\omega })^2+\overline{k}_u(\gamma _{u}^{2\omega }){-}l_u(\delta _{u}^{2\omega })^2 {-}\overline{l}_u (\delta _{u}^{2\omega }),\\{} & {} c_{u}^{(E),3\xi }\left( \sum _{g=1}^G\sum _{k=1}^K x_{guk}^{3\xi },\gamma _u^1,\gamma _u^{2\omega },\delta _u^{2\omega },\delta _u^{3\xi }\right) =i_{u}\left( \sum _{g=1}^G\sum _{k=1}^K x_{guk}^{1}\right) ^2+\overline{i}_u\left( \sum _{g=1}^G\sum _{k=1}^K x_{guk}^{1}\right) \\{} & {} \quad +j_u(\gamma _u^1)^2+\overline{j}_{u}(\gamma _u^1)\\{} & {} +k_u(\gamma _{u}^{2\omega })^2 +\overline{k}_u(\gamma _{u}^{2\omega }){-}l_u(\delta _{u}^{2\omega })^2{-}\overline{l}_u (\delta _{u}^{2\omega }){-}m_u(\delta _{u}^{3\xi })^2{-}\overline{m}_u(\delta _{u}^{3\xi });\\{} & {} c_{\tilde{f}}^{s\theta }=n_{\tilde{f}}\left( \sum _{u=1}^U\sum _{k=1}^K x_{u\tilde{f}k}^{s\theta }\right) ^2+\overline{n}_{\tilde{f}}\left( \sum _{u=1}^U\sum _{k=1}^K x_{u\tilde{f}k}^{s\theta }\right) ,\\{} & {} c_u^{1}=r_u(\gamma _u^{1})^2+\overline{r}_u\gamma _u^{s\theta },\;c_u^{2\omega }=r_u(\gamma _u^{2\omega })^2 +\overline{r}_u\gamma _u^{2\omega };\\{} & {} c_u^{(D),2\omega }=q_u(\delta ^{2\omega })^2+\overline{q}_u\delta ^{2\omega },\;c_u^{(D),3\xi } =q_u(\delta ^{3\xi })^2+\overline{q}_u\delta ^{3\xi }. \end{aligned}$$

For the numerical setting, we consider the coefficients for the cost functions involved in the formulation as in Table 2.

**Table 2:** Coefficients for the cost functions involved in the mathematical formulation

Description	Numerical data
$a_{gu}$ and $\overline{a}_{gu}$: Coefficients of the transmission cost of services from user *g* to the controller UAV *u* both in the first, the second stage under scenario $\omega$ and third stage under scenario $\xi$ ($c_{gu}^1$, $c_{gu}^{2\omega }$, $c_{gu}^{3\xi }$)	$a_{11}=\overline{a}_{11}=0.1$, $a_{12}=\overline{a}_{12}=0.2$, $a_{21}=\overline{a}_{21}=0.1$, $a_{22}=\overline{a}_{22}=0.2$, $a_{31}=\overline{a}_{31}=0.1$, $a_{32}=\overline{a}_{32}=0.2$
$d_{uf}$ and $\overline{d}_{uf}$: Coefficients of the transmission cost of the service requests from controller UAV *u* to any UAV $f\in {\mathcal {F}}_3$ in the first stage, the second stage under scenario $\omega$ and third stage under scenario $\xi$, ($c_{uf}^1$, $c_{uf}^{2\omega }$, $c_{uf}^{3\xi }$)	$d_{1\hat{1}}=0.2$, $d_{1\hat{2}}=0.1$, $d_{1\tilde{1}}=0.1$, $d_{1\tilde{2}}=0.2$, $d_{2\hat{1}}=0.2$, $d_{2\hat{1}}=0.2$, $d_{2\tilde{1}}=0.1$, $d_{2\tilde{2}}=0.2$, $\overline{d}_{1\hat{1}}=0.2$, $\overline{d}_{1\hat{2}}=0.1$, $\overline{d}_{1\tilde{1}}=0.1$, $\overline{d}_{1\tilde{2}}=0.2$, $\overline{d}_{2\hat{1}}=0.2$, $\overline{d}_{2\hat{2}}=0.1$, $\overline{d}_{2\tilde{1}}=0.1$, $\overline{d}_{2\tilde{2}}=0.2$
$g_{f}$ and $\overline{g}_{f}$: Coefficients of the execution cost of requested services to the UAV $f\in {\mathcal {F}}_3$ in the first stage, the second stage under scenario $\omega$ and third scenario under scenario $\xi$, ($c_{f}^{(E),1}$, $c_{f}^{(E),2\omega }$, $c_{f}^{(E),3\xi }$ )	$g_{\hat{1}}=0.2$, $g_{\hat{2}}=0.1$, $g_{\tilde{1}}=0.1$, $g_{\tilde{2}}=0.2$ $\overline{g}_{\hat{1}}=0.2$, $\overline{g}_{\hat{2}}=0.1$, $\overline{g}_{\tilde{1}}=0.1$, $\overline{g}_{\tilde{2}}=0.2$
$n_{\tilde{f}}$ and $\overline{n}_{\tilde{f}}$: Coefficients of the cost due to add new UAVs $\tilde{f}\in \tilde{{\mathcal {F}}}$ at the highest level of the network in the first stage, the second stage under scenario $\omega$ and third stage under scenario $\xi$ ($c_{\tilde{f}}^{1}$, $c_{\tilde{f}}^{2\omega }$, $c_{\tilde{f}}^{3\xi }$)	$n_{\tilde{1}}=\overline{n}_{\tilde{1}}=0.1$, $n_{\tilde{2}}=\overline{n}_{\tilde{2}}=0.2,$
$r_u$ and $\overline{r}_u$: Coefficients of the cost due to add additional capacity to the controller UAV *u*, both in the first and the second stage under scenario $\omega$ ($c_{u}^{1}$, $c_{u}^{2\omega }$)	$r_1=r_2=\overline{r}_1=\overline{r}_2=0.2$
$q_u$ and $\overline{q}_u$: Coefficients of the cost due to reduce capacity to the controller UAV *u*, both in the second stage under scenario $\omega$ and third stage under scenario $\xi$ ($c_{u}^{(D),2\omega }$, $c_{u}^{(D),3\xi }$)	$q_1=q_2=\overline{q}_1=\overline{q}_2=0.05$

Finally, for the coefficients of the management costs of service requests to the controller UAV *u*, in stage 1, in stage 2 under scenario $\omega \in \Omega$ and in stage 3 under scenario $\xi \in \Xi$, we set:

$$\begin{aligned}{} & {} i_1=0.1,\ i_2=0.2,\ \overline{i}_1=0.1,\ \overline{i}_2=0.2, j_1=0.1,\ j_2=0.2,\ \overline{j}_1=0.1,\ \overline{j}_2=0.2,\\{} & {} k_1=0.1,\ k_2=0.2,\ \overline{k}_1=0.1,\ \overline{k}_2=0.2, l_1=-0.05,\ l_2=-0.1,\ \overline{l}_1=-0.05,\ \overline{l}_2=-0.1,\\{} & {} m_1=-0.05,\ m_2=-0.1,\ \overline{m}_1=-0.05,\ \overline{m}_2=-0.1. \end{aligned}$$

Observe that, no constant term appears in the general expression of cost functions described above. Therefore, if there is no flow in a link of the network (from a user or device on the ground to a controller UAV or from a controller UAV to a UAV in the upper tier fleet), the cost of transmission via that link is zero. Similarly, if a UAV does not execute any service, the execution cost for that UAV is zero. The same for the cost needed to add new UAVs and additional capacities and for the cost sustained to reduce capacity. The optimal solutions of the proposed numerical example are computed via the Euler Method (see [18] for a detailed description) using the Matlab program on an HP laptop with an AMD compute cores 2C+3 G processor, 8 GB RAM and are reported in Tables 3 and 4.

**Table 3:** Optimal solutions: $x_{guk}^{s\theta *}$ and $y_{ufk}^{s\theta *}$, for all $g,\ u,\ k,\ \hat{f},\ \tilde{f},\ s,\ \theta$

Variables	Stage	Scenarios		$u=1$	$u=2$	Variables		$u=1$	$u=2$
$x_{guk}^{1*}$	$s=1$	*I*	$$\begin{array}{c} g=1 \\ g=2 \\ g=3\end{array}$$	$$\begin{array}{c}1.32\\ 1.32\\ 3.99\end{array}$$	$$\begin{array}{c}0.67\\ 0.67\\ 2.00\end{array}$$	$y_{uf}^{1*}$	$$\begin{array}{c}\hat{f}=1\\ \hat{f}=2\\ \tilde{f}=1\\ \tilde{f}=2\end{array}$$	$$\begin{array}{c}1.24\\ 0.69\\ 1.81\\ 0.71\end{array}$$	$$\begin{array}{c}2.82\\ 1.73\\ 0.75\\ 0.20\end{array}$$
		$\omega _1$	$$\begin{array}{c}g=1\\ g=2\\ g=3\end{array}$$	$$\begin{array}{c}0.00\\ 1.38\\ 0.00\end{array}$$	$$\begin{array}{c}0.00\\ 1.61\\ 0.00\end{array}$$		$$\begin{array}{c}\hat{f}=1\\ \hat{f}=2\\ \tilde{f}=1\\ \tilde{f}=2\end{array}$$	$$\begin{array}{c}0.24\\ 0.66\\ 0.48\\ 0.00\end{array}$$	$$\begin{array}{c}0.00\\ 0.62\\ 0.00\\ 0.00\end{array}$$
$x_{guk}^{2\omega *}$	$s=2$	$\omega _2$	$$\begin{array}{c}g=1\\ g=2\\ g=3\end{array}$$	$$\begin{array}{c}2.64\\ 0.00\\ 4.64\end{array}$$	$$\begin{array}{c}1.35\\ 0.00\\ 2.35\end{array}$$	$y_{uf}^{2\omega *}$	$$\begin{array}{c}\hat{f}=1\\ \hat{f}=2\\ \tilde{f}=1\\ \tilde{f}=2\end{array}$$	$$\begin{array}{c} 1.37\\ 3.08\\ 1.98\\ 0.84\end{array}$$	$$\begin{array}{c}0.78\\ 1.89\\ 0.79\\ 0.25\end{array}$$
		$\omega _3$	$$\begin{array}{c}g=1\\ g=2\\ g=3\end{array}$$	$$\begin{array}{c}6.44\\ 5.11\\ 6.44\end{array}$$	$$\begin{array}{c}5.55\\ 4.88\\ 5.55\end{array}$$		$$\begin{array}{c}\hat{f}=1\\ \hat{f}=2\\ \tilde{f}=1\\ \tilde{f}=2\end{array}$$	$$\begin{array}{c} 2.66\\ 3.33\\ 6.58\\ 5.42\end{array}$$	$$\begin{array}{c} 2.33\\ 2.66\\ 5.92\\ 5.08\end{array}$$
		$\xi _1$	$$\begin{array}{c}g=1\\ g=2\\ g=3\end{array}$$	$$\begin{array}{c}0.69\\ 1.36\\ 2.02\end{array}$$	$$\begin{array}{c}0.31\\ 0.64\\ 0.98\end{array}$$		$$\begin{array}{c}\hat{f}=1\\ \hat{f}=2\\ \tilde{f}=1\\ \tilde{f}=2\end{array}$$	$$\begin{array}{c} 0.73\\ 1.80\\ 1.12\\ 0.41\end{array}$$	$$\begin{array}{c} 0.37\\ 1.09\\ 0.40\\ 0.05\end{array}$$
		$\xi _2$	$$\begin{array}{c}g=1\\ g=2\\ g=3\end{array}$$	$$\begin{array}{c}1.34\\ 0.67\\ 3.34\end{array}$$	$$\begin{array}{c}0.66\\ 0.32\\ 1.66\end{array}$$		$$\begin{array}{c}\hat{f}=1\\ \hat{f}=2\\ \tilde{f}=1\\ \tilde{f}=2\end{array}$$	$$\begin{array}{c} 0.99\\ 2.31\\ 1.47\\ 0.58 \end{array}$$	$$\begin{array}{c} 0.54\\ 1.40\\ 0.56\\ 0.13 \end{array}$$
		$\xi _3$	$$\begin{array}{c}g=1\\ g=2\\ g=3\end{array}$$	$$\begin{array}{c}2.57\\ 0.57\\ 4.57\end{array}$$	$$\begin{array}{c}1.43\\ 0.43\\ 2.43\end{array}$$		$$\begin{array}{c}\hat{f}=1\\ \hat{f}=2\\ \tilde{f}=1\\ \tilde{f}=2\end{array}$$	$$\begin{array}{c} 1.47\\ 3.27\\ 2.09\\ 0.89 \end{array}$$	$$\begin{array}{c} 0.89\\ 2.12\\ 0.94\\ 0.32 \end{array}$$
$x_{guk}^{3\xi *}$	$s=3$	$\xi _4$	$$\begin{array}{c}g=1\\ g=2\\ g=3\end{array}$$	$$\begin{array}{c}0.69\\ 1.36\\ 2.02\end{array}$$	$$\begin{array}{c}0.31\\ 0.64\\ 0.97\end{array}$$	$y_{uf}^{3\xi *}$	$$\begin{array}{c}\hat{f}=1\\ \hat{f}=2\\ \tilde{f}=1\\ \tilde{f}=2\end{array}$$	$$\begin{array}{c}0.73\\ 1.80\\ 1.12\\ 0.41\end{array}$$	$$\begin{array}{c}0.38\\ 1.08\\ 0.41\\ 0.05\end{array}$$
		$\xi _5$	$$\begin{array}{c}g=1\\ g=2\\ g=3\end{array}$$	$$\begin{array}{c}2.62\\ 2.62\\ 3.69\end{array}$$	$$\begin{array}{c}1.37\\ 1.37\\ 2.04\end{array}$$		$$\begin{array}{c}\hat{f}=1\\ \hat{f}=2\\ \tilde{f}=1\\ \tilde{f}=2\end{array}$$	$$\begin{array}{c}1.79\\ 3.74\\ 2.55\\ 1.13\end{array}$$	$$\begin{array}{c}1.06\\ 2.26\\ 1.08\\ 0.39\end{array}$$
		$\xi _6$	$$\begin{array}{c}g=1\\ g=2\\ g=3\end{array}$$	$$\begin{array}{c}4.77\\ 4.11\\ 6.11\end{array}$$	$$\begin{array}{c}2.22\\ 1.88\\ 2.88\end{array}$$		$$\begin{array}{c}\hat{f}=1\\ \hat{f}=2\\ \tilde{f}=1\\ \tilde{f}=2\end{array}$$	$$\begin{array}{c}3.16\\ 4.33\\ 5.1\\ 2.4\end{array}$$	$$\begin{array}{c}1.83\\ 1.66\\ 2.43\\ 1.07\end{array}$$
		$\xi _7$	$$\begin{array}{c}g=1\\ g=2\\ g=3\end{array}$$	$$\begin{array}{c}5.73\\ 5.53\\ 5.73\end{array}$$	$$\begin{array}{c}2.58\\ 2.48\\ 2.58\end{array}$$		$$\begin{array}{c}\hat{f}=1\\ \hat{f}=2\\ \tilde{f}=1\\ \tilde{f}=2\end{array}$$	$$\begin{array}{c}3.28\\ 4.56\\ 6.20\\ 2.95\end{array}$$	$$\begin{array}{c}1.71\\ 1.44\\ 3.08\\ 1.39\end{array}$$
		$\xi _8$	$$\begin{array}{c}g=1\\ g=2\\ g=3\end{array}$$	$$\begin{array}{c}5.65\\ 5.65\\ 5.65\end{array}$$	$$\begin{array}{c}2.56\\ 2.56\\ 2.56\end{array}$$		$$\begin{array}{c}\hat{f}=1\\ \hat{f}=2\\ \tilde{f}=1\\ \tilde{f}=2\end{array}$$	$$\begin{array}{c}3.29\\ 4.52\\ 6.18\\ 2.96\end{array}$$	$$\begin{array}{c}1.71\\ 1.47\\ 3.13\\ 1.39\end{array}$$

**Table 4:** Optimal solutions: $\gamma _u^{1*}$, $\gamma _u^{2\omega *}$, $\delta _u^{2\omega *}$, $\delta _u^{3\xi *}$, for all *u*, $\omega \in \Omega$, $\xi \in \Xi$

Variables	Stages	Scenarios	$u=1$	$u=2$	Variables	$u=1$	$u=2$	Lagrange multipliers
$\gamma _u^{1*}$	$s=1$	*I*	3	1.55		//	//	$\lambda ^{1*}=0.00$
$\gamma _u^{2\omega *}$	$s=2$	$\omega _1$	0.00	0.00	$\delta _u^{2\omega *}$	3	1.55	$\lambda ^{2\omega _1*}=0.00$
		$\omega _2$	0.00	0.00		1.55	0.00	$\lambda ^{2\omega _2*}=0.00$
		$\omega _3$	10.00	7.44		0.00	0.00	$\lambda ^{2\omega _3*}=0.00$
	$s=3$	$\xi _1$	//	//	$\delta _u^{3\xi *}$	0.00	0.00	$\lambda ^{3\xi _1*}=0.00$
		$\xi _2$	//	//		1.00	0.00	$\lambda ^{3\xi _2*}=0.00$
		$\xi _3$	//	//		0.00	0.00	$\lambda ^{3\xi _3*}=0.00$
		$\xi _4$	//	//		1.00	9.00	$\lambda ^{3\xi _4*}=0.00$
		$\xi _5$	//	//		1.00	9.00	$\lambda ^{3\xi _5*}=0.00$
		$\xi _6$	//	//		1.00	9.00	$\lambda ^{3\xi _6*}=0.00$
		$\xi _7$	//	//		0.99	8.37	$\lambda ^{3\xi _7*}=0.00$
		$\xi _8$	//	//		1.00	8.29	$\lambda ^{3\xi _8*}=0.00$

**Fig. 6:**
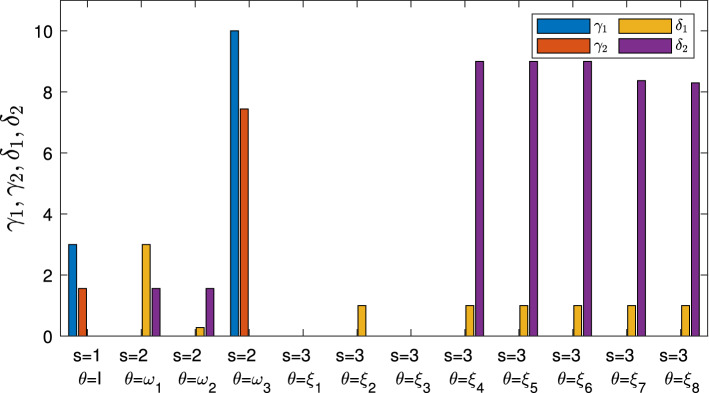
Optimal solutions: additional and reduced capacities ($\gamma _{u}^{s\theta }$ and $\delta _{u}^{s\theta }$)

**Fig. 7:**
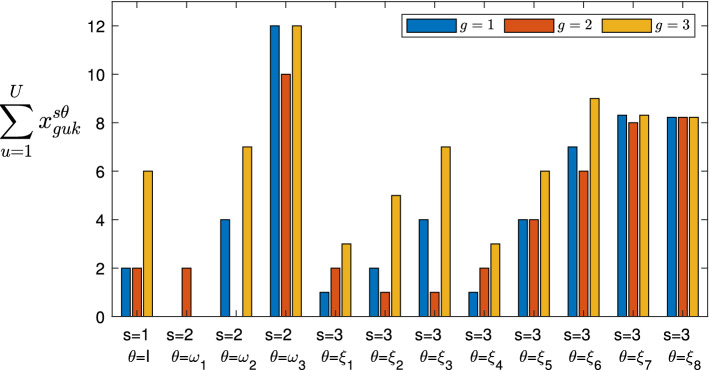
Amount of data transmitted by each user or device

**Fig. 8:**
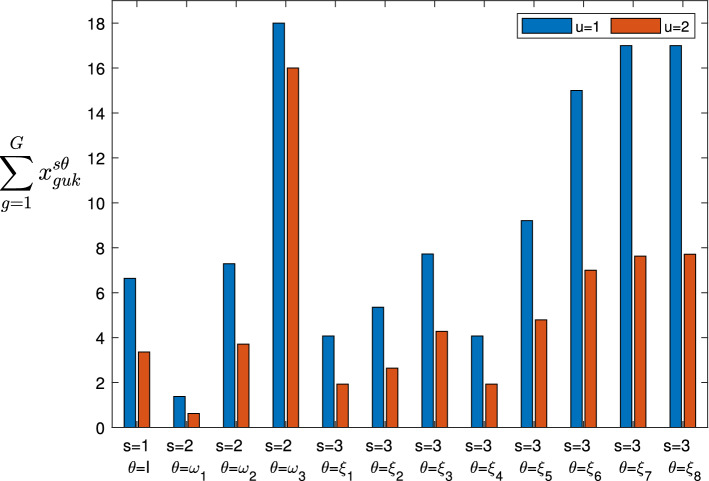
Controller UAVs utilization

**Fig. 9:**
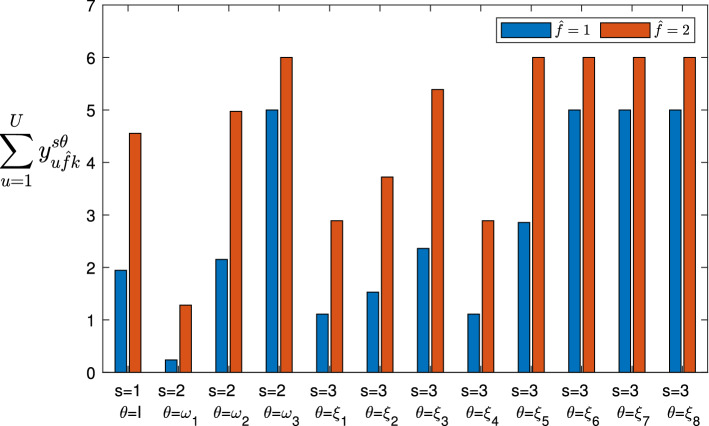
Pre-existing UAVs utilization

From the obtained optimal solution we can observe that, despite the demands for service requested by user or device on the ground is not high, it is convenient to add capacities to the controller UAVs. Moreover, it is suitable to add the maximum allowed capacity on the first controller UAV and to use also additional UAVs, in order to be prepared for the following stages. We underline that during each of the scenarios of the second stage, the data transmitted during the first preparedness stage are used to satisfy all the requests. Furthermore, note that during the first scenario of stage 2 ($\omega _1\equiv I$) no additional capacities are used, and rather, they are reduced (because, obviously, the only scenario that follows $\omega _1$ is the initial scenario $\xi _1\equiv I$). Instead, during the second scenario of stage 2, when a landslide occurs, only a portion of added capacities are reduced (because some of them are used also during the following scenarios in stage 3) and during the third scenario of stage 2, that is when an earthquake occurs, the maximum allowed capacity of the first controller UAV and much of the additional capacity of the second are used (see Fig. 6) because we assumed that the earthquake is most serious event. The same occurs for the additional UAVs belonging to the fleet at the upper tier of the network.

In stage 1 and stage 2 all the requests are satisfied. In the first three scenarios of stage 3 all the requests are satisfied, too, while for the rest of the scenarios it is convenient to satisfy only a portion of service requests (see Fig. 7).

Except for stage 1, as shown from Figs. 8, 9 and 4, the utilization of both the controller and the pre-existing and additional UAVs follows the same increasing trend of requests and, hence, the same trend of the gravity of each scenario. Indeed, when an earthquake occurs most of the resources are fully utilized, while for the normalcy situation resources are underutilized. Moreover, we observe that the controller UAV $u=1$ is used more than the second, the second pre-existing UAV ($\hat{f}=2$) is used more than the first and the additional UAV $\tilde{f}=1$ is used more than the second one. These aspects are justified by the cost functions Fig. 6.

## Summary and conclusions

Disaster management is of major importance since the number of disasters and their frequency have been increasing, with the negative impacts on societies and economies rising. Disasters can be slow-onset or sudden-onset and be “natural,” or “man-made,” with climate change adding to the severity of various disasters, notably, hurricanes and floods as well as droughts. Managing a disaster entails the identification of scenarios in order to plan for, respond to and recover from such crises. The understanding of the objectives of organizations, both public and private ones, involved in disaster management is also paramount, along with the optimization of resource utilization.

In an increasingly connected world, the provision of services through advanced technologies, such as 5th Generation networks, is becoming essential, and offers opportunities for enhanced disaster management. The need for arming service providers with mathematical tools to help them to manage resources optimally in disaster situations has led us to develop, in this paper, a multi-stage stochastic optimization model based on a three-tiered network. The model consists of users and devices on the ground, requesting 5G services of a fleet of controller UAVs that, in turn, manage and send the requests to a fleet of UAVs, organized as a FANET, which execute them. Such UAVs are connected to each other via the 5G network, which is fast, stable and secure. The service providers can receive data to their UAVs in real-time from devices or sensors located on the ground. In this framework, we mathematically capture three phases of disaster management: preparedness, response and recovery/reconstruction. In the first stage of our model, which represents the preparedness phase, service providers make predictions about possible disaster scenarios that could occur in the second stage, which represents the critical response phase. This phase is followed by a third stage, that of recovery/reconstruction.

Service providers solve a three-stage stochastic optimization problem, where, in the first stage, they seek to maximize the quantity of provided 5G services, associating a priority to each of them, and determine how to best manage the UAVs of the upper-tier fleet, while minimizing the overall costs associated with the management of service requests and those of transmission and execution of services. In this stage, service providers can decide whether or not to provide more services than required in order to satisfy the request, which could grow unexpectedly, in the subsequent stage. Hence, they can decide to increase the controller UAV capacities and to add additional UAVs to the pre-existing fleet; however, by incurring additional costs.

In the second stage, with the acknowledgment of the gravity of the disaster, service providers, in addition to pursuing the objectives of the previous stage, decide whether to further increase the capacity of the controller UAVs (in the event of more severe disasters) and to add other vehicles to the fleet, or whether to reduce the capacity of controller UAVs (in the case of less severe scenarios). Moreover, in the second stage the penalty due to the unmet demand for services is also minimized. In the third stage, the effects of the disaster can cease, with a return to a normal situation; a reduction in severity may occur or the disaster effects can persist. Hence, in this stage, service providers, pursuing the same objectives as in the previous two stages, can decide whether to further reduce the capacity of the controller UAVs.

Each of the first, second and third stage optimization problems is subject to a multitude of constraints, including: conservation, demand, capacity and budget constraints. Following classical stochastic optimization theory, these three constrained optimization problems are formulated and solved as a stochastic multi-stage constrained optimization model, which is, in turn, formulated as a finite-dimensional variational inequality problem. Existence and uniqueness results for the solution to the variational inequality problem are also provided. In addition, a detailed numerical example, with three scenarios in the second stage and eight scenarios in the third stage is provided, along with the computed optimal values of over 200 variables, in order to illustrate some of the key aspects of the proposed model.

The obtained results provide us with some managerial insights, since it may be useful to use the proposed formulation not only to determine the optimal distribution of flows, but also the optimal management of resources. Indeed, the optimal solution shows that, at the first stage, it is convenient to add (and use) some new UAVs to the fleet executing the services and some additional capacities to the controller UAVs, in order to be prepared for the following stages. At the second stage, if no disaster event or a minor disaster (such as a little landscape) occurs, it is suitable to use only some additional UAVs (to ensure that the demand is met) and to reduce the capacities on the controller UAVs (because they will not be used even during the third stage, when the requests of services are assumed to be less than that of the second stage). While, if a serious scenario (such as an earthquake) occurs, the provider needs to use all the additional resources in order to satisfy all the requests of services. Therefore, it is clear that the mathematical formulation is efficient and adapts to reality and to different types of scenarios (even with different probability of occurrence) in the three stages. Furthermore, using the proposed model, as a simulation tool, the providers can obtain useful information, especially on the use of resources. Providers could, hence, both establish in advance whether to buy new resources (UAVs and capacities) and instantly decide if and which resources are convenient to use, to add or to reduce, with the main objective of satisfying all the requests for the necessary services during a disastrous event.

In our future work, we intend to do additional sensitivity analysis and to discuss the computational complexity of the problem. Moreover, we are going to study a more comprehensive model, in which we introduce four stages (Mitigation, Preparedness, Response, and Recovery) and a bigger area to be covered (intended as a union of small areas) in which we investigate the impacts of the size of the area and a more general case of multi-hop communication between UAVs in the same network. Therefore, we intend to test the proposed formulation by solving numerical examples on large and real instances.

## Data Availability

The datasets generated during and/or analysed during the current study are available from the corresponding author on reasonable request.
